# Regional and national estimates of children affected by all-cause and COVID-19-associated orphanhood and caregiver death in Brazil, by age and family circumstance: a modeling study

**DOI:** 10.1016/j.lana.2025.101252

**Published:** 2025-09-29

**Authors:** Nicholas Steyn, H. Juliette T. Unwin, Jamie Ponmattam, Andrés Villaveces, Luiza Martins, Lorraine Sherr, Alexandra Blenkinsop, Elizaveta Semenova, Alice Stuart-Brown, André Victor Ribeiro Amaral, Oliver Ratmann, Ricardo Parolin Schnekenberg, Lucie Cluver, Susan Hillis, Laura Rawlings, Lorena Barberia, Andrea Santos Souza, Marcia C. Castro, Seth Flaxman

**Affiliations:** a Department of Statistics, University of Oxford, Oxford, United Kingdom; b School of Mathematics, University of Bristol, Bristol, United Kingdom; c School of Public Health, Imperial College London, London, United Kingdom; d Department of Global Health and Population, Harvard T H Chan School of Public Health, Boston, MA, United States of America; e Department of Epidemiology, University of North Carolina at Chapel Hill, Chapel Hill, NC, United States of America; f Federal University of Minas Gerais, Minas Gerais, Brazil; g Institute of Global Health, University College London, London, United Kingdom; h Department of Mathematics, Imperial College London, London, United Kingdom; i School of Mathematical Sciences, University of Southampton, Southampton, United Kingdom; j Institute of Neurology, University College London, London, United Kingdom; k Department of Social Policy and Intervention, University of Oxford, Oxford, United Kingdom; l Global Reference Group on Children Affected by Crisis, University of Oxford, Oxford, United Kingdom; m World Bank Group, Washington, DC, United States of America; n Department of Political Science, University of São Paulo, São Paulo, Brazil; o Ministério Público do Estado de São Paulo, São Paulo, Brazil; p Department of Computer Science, University of Oxford, Oxford, United Kingdom

**Keywords:** Orphanhood, Brazil, COVID-19, Excess mortality, Caregiver death

## Abstract

**Background:**

Orphanhood and caregiver death can have severe consequences for children. Timely and accurate data can guide policy, particularly during health crises like COVID-19. The aim of our study is to present national and subnational analysis of both all-cause and COVID-19-associated orphanhood and caregiver death in Brazil and compare our model outputs with bespoke administrative datasets.

**Methods:**

We use publicly available national datasets to estimate the number of Brazilian children experiencing parent and caregiver loss due to all causes and COVID-19 in 2020–2021.

**Findings:**

An estimated 1,300,000 (95% uncertainty interval, 1,190,000, 1,430,000) children in Brazil experienced loss of one or multiple parents and/or co-residing caregivers. 673,000 (652,000, 690,000) were estimated to have lost one or both parents, of which 149,000 (144,000, 154,000) were COVID-19 associated; 635,000 (534,000, 758,000) children were estimated to have lost a co-residing grandparent or other kin, of which 135,000 (85,900, 199,000) were COVID-19 associated. Orphanhood varied substantially across states, with the rate of all cause parental orphanhood highest in Roraima at 17.5 (15.6, 20.6) per 1000 children and the lowest in Santa Catarina at 9.5 (8.7, 10.4) per 1000 children. COVID-19-associated orphanhood was also unevenly distributed, with Mato Grosso experiencing the greatest rate, at 4.4 (3.9, 5.3) per 1000 children, while Pará experienced the lowest rate of 1.4 (1.2, 1.8) per 1000 children. Comparisons with limited data from Brazil’s civil registry offices and (manually reviewed death certificates in Campinas found a similar demographic distribution of orphanhood. However, our estimates suggested that administrative sources undercount orphanhood.

**Interpretation:**

Our findings highlight the extent of orphanhood in Brazil and large inequalities between states. Comparisons between administrative data and model estimates show similar temporal patterns and proportions of maternal and paternal orphanhood but different magnitudes. This suggests that strengthening vital registration systems can put children at the center of public health responses globally.

**Funding:**

This study was funded by “Building Global Public Health Capacity to Link Real-Time Modelling Data on COVID-19-associated Orphanhood and Caregiver Deaths to Inform Prevention, Preparedness and Protection from COVID-19 consequences” (2023 CDC/WHO grant) and the 10.13039/100014000Moderna Charitable Foundation.

**Research in context**

**Evidence before this study**

We searched PubMed for (“COVID-19” OR “SARS-COV-2”) AND “ORPHANHOOD”, returning 14 research articles published between 2020 and 2024. One publication produced estimates of maternal COVID-19-associated orphanhood in Brazil, three publications produced national-level estimates of COVID-19-associated orphanhood in Brazil using global methods, two publications considered COVID-19-associated orphanhood in other regions, while the remaining publications discussed the implications of COVID-19-associated orphanhood but did not produce estimates. The COVID-19 pandemic, and evidence from previous pandemics, including HIV/AIDS and Ebola virus disease, demonstrate extensive consequences of parent and caregiver death during pandemics.

**Added value of this study**

Our study expands and refines previous work on global analyses of COVID-19-associated orphanhood, presenting the first national and subnational analysis of both all-cause and COVID-19-associated orphanhood (both maternal and paternal) and caregiver death in Brazil. We use detailed demographic, mortality, and household survey data to produce age, sex, and region-specific estimates. We compare our model results against two administrative datasets and quantify under-ascertainment. We also estimate loss of co-residing elderly caregivers.

**Implications of all the available evidence**

All-cause orphanhood and caregiver death in Brazil are substantial and unevenly distributed, highlighting structural inequalities. COVID-19-associated orphanhood accounts for a large share of these losses, underscoring the need for crisis-prepared social protection systems. Our findings support legislative action and policy reform to identify, support, and monitor bereaved children. Strengthening civil registration and vital statistics systems would help with timely public health responses and for reaching vulnerable children.

## Introduction

Orphanhood (death of one or both parents) and caregiver death among children aged 0–17 years, across causes of parent and caregiver death, can have severe immediate and life-long consequences for children. Over 15 years ago, the World Health Organisation (WHO) classified orphanhood as an adverse childhood experience (ACE), linked to long-term mental health risks.^1^ As an ACE, parent or caregiver loss may have lifelong consequences, including increased risks of suicide, post-traumatic stress disorder, violence, insecure housing, institutionalization, poverty, and chronic and infectious diseases.^2^^,^^3^ Children experiencing orphanhood (“orphans”) are defined as those aged 0–17 whose father or mother died during childhood or adolescence, while the term “double orphan” refers to children who have lost both parents.^4^ We also consider the loss of co-residing elderly kin (defined as individuals aged 60+ living in the same household), who often play a critical role in childcare and support.^5^^,^^6^

In spite of its acute and enduring lifelong consequences, the threat of all-cause orphanhood has been largely invisible as a major global public health priority. However, orphanhood is a recognized threat to children in the context of disease epidemics, including HIV/AIDS,^7^^,^^8^ COVID-19^6^ and Ebola.^9^ Given the enduring impact of all-cause orphanhood on children and the context of escalating polycrisis threats to children and families, it is helpful to understand the nature and extent of the burden, geographical and regional variances, and the drivers not only of orphanhood but also of co-residing caregiving loss among children and the context in which the caregiver(s) are lost, thus facilitating better provisioning of support and care for affected children.^6^

A variety of interventions to improve the lives of orphans and vulnerable children have been studied,^10^ with promising evidence for social protection combined with parenting support for surviving caregivers.^11^ Delivering protection and support for children orphaned from any cause is only possible if bereaved children can be identified and linked to care in a timely manner. Brazil is unique in the world in requiring a field on death certificates recording child dependents in the household. This simple data gives the possibility of swiftly identifying children affected by orphanhood and caregiver death, so that their health, social, and economic needs can be assessed.^12^

Andrea Santos Souza (a public prosecutor and co-author), aware of the scale of COVID-19-associated orphanhood in Brazil,^6^ obtained death certificates to systematically identify and link COVID-19 orphans to economic support in Campinas (a municipality in São Paulo State). Administrative data on orphanhood is rare globally, so its existence in Brazil is a unique opportunity both to provide support to orphaned children and to compare statistical models for estimating orphanhood with observed data. Furthermore, marked subnational disparities in COVID-19-associated cases and deaths in Brazil^13^ have been linked to differing public health interventions, levels of preparedness^14^ and pre-existing inequities.^15^ The degree to which all-cause orphanhood and the subset of COVID-associated orphanhood distributions also reflect these subnational public health disparities has not been described.

The aim of our study is to present national and subnational analysis of both all-cause and COVID-19-associated orphanhood and caregiver death in Brazil. We also compare our model outputs against bespoke administrative datasets to quantify potential under-ascertainment of orphanhood.

## Methods

We adapted existing methods^6^ to estimate all-cause and COVID-19-associated orphanhood and caregiver death in children aged 0 to 17 in Brazil at the state and national level for 2020 and 2021. Our calculations rely on estimates of fertility rates, household composition, and excess mortality in adults. To minimise the impact of reporting biases, we use excess mortality to quantify COVID-19-associated deaths. We additionally considered the impact of deaths of co-residing individuals aged 60 years and older (60+) as a proxy for the loss of custodial grandparents and other older kin. All our estimates are structured by age-group (in 10-year groups limited by the disaggregation of the all-cause mortality data) and sex, with further disaggregation by month, federative unit (26 states and 1 federal district), and current age-of-child where necessary. As in,^16^ live births and population data were used to produce estimates of female fertility,^17^^,^^18^ while accounting for underreporting (full details in Supplementary material section 1.2.2). The Pesquisa Nacional de Saúde (PNS–National Health Survey, 2019) was used to estimate fertility rates for men aged 18+ and the household composition of adults 60+ (full details in Supplementary material section 1.2.3). From 2010 onwards, the age of the father was included in some live births records. We use these data in Supplementary material section 2.3 to validate our estimates. All-cause mortality estimates for Brazil between January 2020 and December 2021, used in this analysis, are available in Ponmattam et al. (2024). We followed The Lancet’s GATHER checklist while performing and writing this study.

The number of children experiencing the death of a mother and/or a father was estimated as the number of children currently aged 0 to 17 who were born to parents who died, assuming that age-structured fertility rates are independent of the propensity for a given parent to die. The estimate was performed at the state, parent age-group, parent sex, and year-of-death level, and additionally at the age-of-child or month-of-death level where necessary, before being aggregated. Estimates of children who experienced grandparent or older kin loss were created by summing the number of children living in households where a single person aged 60+ died, where multiple people aged 60+ died, and where someone aged 60+ died and where no adults aged 18–59 were living in the household (indicating the loss of a direct caregiver).

Uncertainty in live-births reporting, survey data used to estimate male fertility and older persons household composition, and mortality was accounted for via bootstrapping.^19^ Individual-level uncertainty in female fertility estimates was accounted for by assuming Poisson noise about the population-level bootstrap samples, while individual-level uncertainty in male fertility and household composition was derived directly from the survey data. The mean and the 2.5th and 97.5th quantiles of the bootstrap samples provided our central estimates and uncertainty intervals. Full details are provided in Supplementary material section 1.2.

### All-cause and excess mortality

Expected all-cause mortality was predicted using a Poisson generalized linear mixed model that was trained on 2013–2019 historical mortality data obtained from Brazil’s Mortality Information System.^20^ The fixed parameters were 1-year lagged all-cause mortality rates, and month and age group indicators; states were included as random parameters so that each state had a random intercept and slope on the year variable. Each sex was modeled separately. Excess mortality is estimated as the number of observed deaths which are in excess of the expected number for a given time and place.^20^ Our time period is one in which COVID-19 was a major cause of death, likely accounting for the vast majority of excess mortality.^20^ Excess mortality is a more accurate representation of the overall impact of the COVID-19 pandemic than COVID-19-reported deaths,^21^ which were under-reported during the ongoing epidemic when health systems were overloaded,^15^ thus enabling us to come closer to the true magnitude of children whose parents or caregivers died during the COVID-19 pandemic. Estimates of all-cause and excess mortality by month, age-group, sex, and state (and federal district) were obtained for the period from January 2020 through December 2021.^20^ We report estimates associated with excess deaths as “COVID-19-associated orphanhood”, while estimates associated with all-cause mortality are reported as “all-cause orphanhood”.

Confidence intervals for monthly data were not available, and as such we did not further account for uncertainty in monthly excess mortality estimates in our analyses. Monthly data were only used when presenting trends in orphanhood results by month in 2020 and 2021.

### Estimating orphanhood

Samples of orphanhood resulting from the death of a parent in a specific age-group, sex, state, and year are constructed by first sampling the number of deaths (excess or all-cause) that occurred in adults in this group. Then, for each parent that died, we sample (from estimates of female or male cumulative child fertility rates) the number of children aged 0 to 17 that they left behind. Totaling all children left behind (in each age-group, sex of parent, state, and year combination) produces a single sample from the distribution of orphanhood for that combination.

Repeating this procedure produces a collection of samples of orphanhood that approximate the distribution of orphanhood. Our central estimates are the average of these samples, while the 2.5th and 97.5th quantiles define our 95% uncertainty intervals (U.I.). Aggregated estimates of orphanhood (for example, total orphanhood, or orphanhood by state) were calculated by summing over the relevant samples and then calculating the average and quantile values.

To account for double orphanhood (the loss of both parents) we estimated the number of male-female couples where both have died (full details are provided in Supplementary material section 1.2.4). We do not explicitly account for adult mortality pre-2020, so our definition of double orphanhood includes pre-existing single orphans who became double orphans in 2020 or 2021.

### Estimating the loss of co-residing grandparent or older kin

We used the PNS 2019 survey to consider household-based estimates of orphanhood, allowing us to estimate the number of children that lost a co-residing grandparent or older kin. We used mortality estimates to assign excess or all-cause deaths to individuals aged 60+ that featured in the survey with probability E/N, where E is the number of excess deaths that occurred in individuals in a specific age-group, sex, state, and year, and N is the corresponding population size.

We then used the R *survey* package^22^ to estimate the total number of children living in households where:

• At least one elderly co-residing person has died (“any”)
• A single elderly co-residing person has died (“single”)
• Multiple elderly co-residing people have died (“multiple”)
• At least one elderly co-residing person has died and there are no adults aged 18–59 (“direct”, representing the loss of a direct caregiver)

The *svytotal* function from the *survey* package in R was used to estimate a survey-weighted mean and standard error for total orphanhood due to the loss of co-residing elderly. This process was repeated 100 times, at each iteration reassigning excess deaths, estimating a new mean and standard error, and taking 10 samples of total orphanhood from the approximately Normal distribution (with the corresponding estimated mean and standard error). This process produced 1000 samples of orphanhood due to the loss of co-residing elderly that accounted for uncertainty in excess mortality and household composition.

To estimate the total number of children experiencing either parental orphanhood or the loss of co-residing grandparents or older kin, we add the estimated number of children experiencing parental orphanhood and the estimated number of children that lost co-residing grandparents or older kin, and subtract the small number of children that experienced both, estimated using unweighted PNS data by state (Supplementary material section 1.2.6).

### Administrative data

We obtained two sources of administrative data to compare representative proportions of our state level estimates with. Arpen-Brasil, which represents Brazil’s Civil Registry offices, used administrative records to link birth and death certificates in 2021 for children aged 6 or under^23^ and shared summary tables of their results. This dataset, released in October 2021, consists of children who experienced orphanhood between 18 March 2020 and 24 September 2021. A total of 12,566 orphans (including maternal, paternal, and double) were identified in this period for children aged 6 or under: n = 12,211 for children whose parents’ death have been recorded as COVID-19 and n = 355 for Severe Acute Respiratory Syndrome (SRAG in Portuguese) deaths. The average age of these children was 2.0 years. While COVID-19 is a likely cause of death for the SRAG deaths, we restrict our analysis to the COVID-19-confirmed deaths.

Co-author Andrea Santos Souza and a team of research assistants manually reviewed all death certificates in 2020 and 2021 listing COVID-19 as the cause of death in Campinas and created a list of affected children 0–17. They uncovered a total of 481 children aged 0–17 listed as child dependents in the household of adults who had died from COVID-19. Of these, 375 children had their age recorded, with a mean age of 11.5 years.

### Role of the funding source

The funders had no role in the study design, collection, analysis, and interpretation of data, writing of the report or decision to submit the paper for publication.

## Results

### All-cause orphanhood

**Fig. 1:**
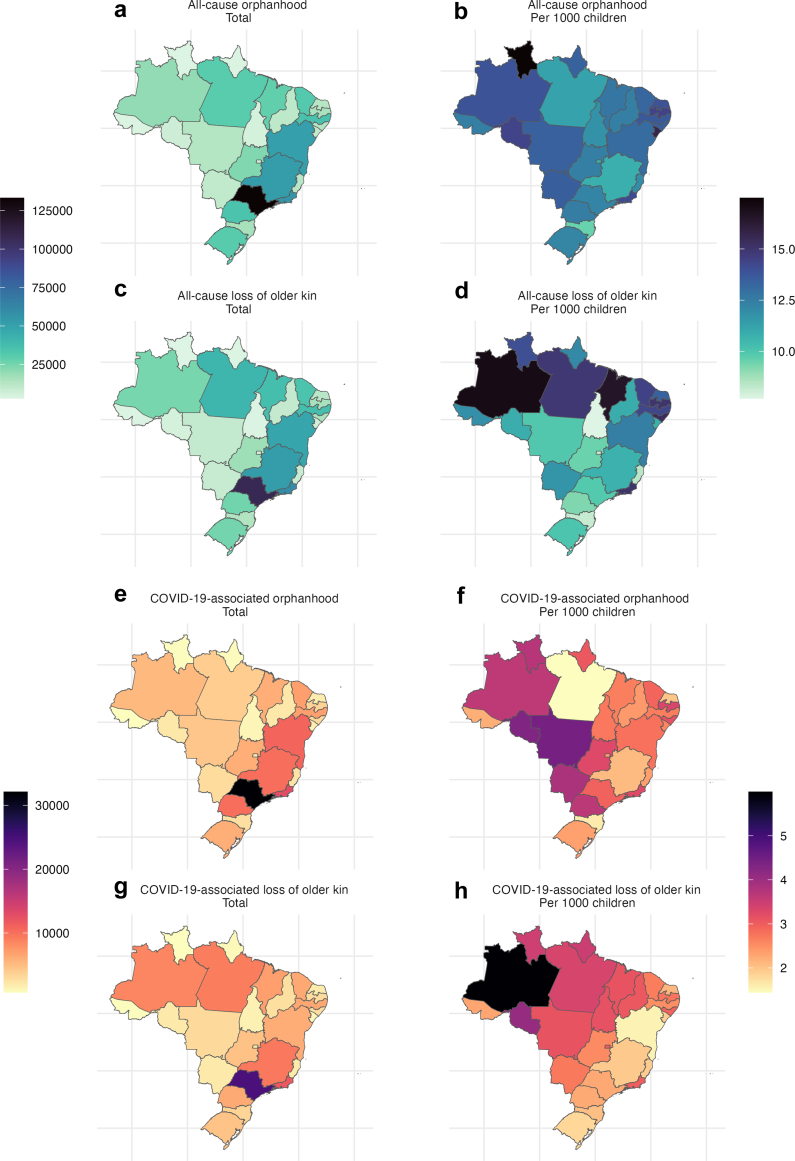
Estimates of (a, b) the number of children experiencing orphanhood due to all causes, (c, d) the number of children that lost a co-residing grandparent or other older kin due to all causes, (e, f) the number of children experiencing orphanhood due to COVID-19-associated causes, and (g, h) the number of children that lost a co-residing grandparent or other older kin due to COVID-19-associated causes. Left-hand figures show absolute estimates, right-hand figures show estimates per 1000 children in the given state. Brazil shapefiles were obtained using the *geobr* R package.^24^

**Fig. 2:**
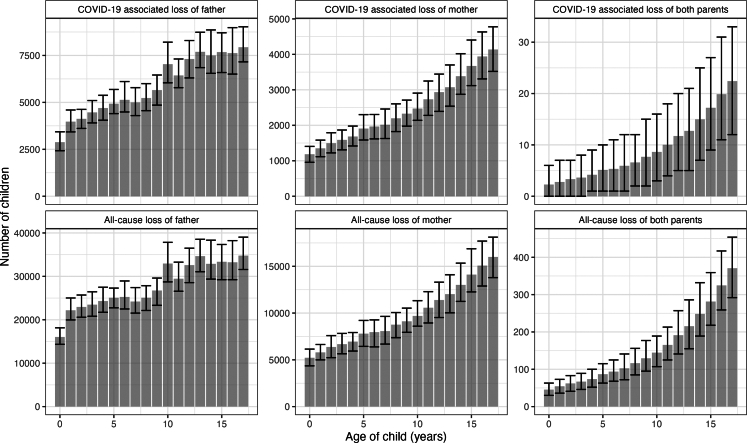
Age distribution of children experiencing orphanhood in Brazil from January 2020 through December 2021 by family circumstance (death of father, death of mother, death of both parents). COVID-19 associated orphanhood is shown in the top panels, all-cause orphanhood is shown in the bottom panels.

We estimate that, from January 2020 through December 2021, a total of 1,300,000 (95% uncertainty interval 1,190,000, 1,430,000) children in Brazil experienced or grand/or grandparent or older kin (two-year incidence). Of these, 673,000 (95% uncertainty interval (U.I.) 652,000–690,000) children lost one or both parents due to any cause of death (Table 1, Fig. 1). Consistent with the global mortality impacts of COVID-19 which affected men disproportionately, and the tendency for men to have children at older ages, paternal orphanhood (496,000 children, or approximately 73.8% of all orphaned children) was estimated to be more frequent than maternal orphanhood (174,000 children, or approximately 25.8% of all orphaned children). The remaining 2730 children (0.4%) were orphaned due to the loss of both parents. Orphanhood was estimated to be greater in older children than younger children, particularly for the loss of mothers and the loss of both parents (Fig. 2).

**Table 1:** Estimates of orphanhood due to any cause of parental death and estimates of the number of children that lost a co-residing grandparent or other older kin.

Federative unit	All-cause orphanhood		All-cause loss of grandparents or older kin		All-cause loss of parent and/or grandparents or older kin	
	Total	Per 1000 children	Total	Per 1000 children	Total	Per 1000 children
Acre	3890 (3470, 4370)	12.5 (11.1, 14)	3700 (1460, 7030)	11.8 (4.7, 22.5)	7540 (5220, 11,100)	24.1 (16.7, 35.5)
Alagoas	13,200 (12,000, 14,800)	13.5 (12.4, 15.2)	14,900 (0, 28,600)	15.4 (0, 29.4)	27,900 (14,100, 41,500)	28.7 (14.5, 42.6)
Amapá	3910 (3410, 4470)	13.4 (11.7, 15.3)	3530 (0, 7580)	12.1 (0, 26)	7360 (3660, 11,600)	25.2 (12.6, 39.7)
Amazonas	20,000 (18,000, 21,800)	13.9 (12.5, 15.1)	24,500 (13,500, 39,500)	17.1 (9.4, 27.5)	44,100 (32,400, 59,700)	30.7 (22.5, 41.5)
Bahia	50,900 (45,500, 56,100)	13.1 (11.7, 14.4)	48,200 (15,100, 102,000)	12.4 (3.9, 26.4)	98,500 (64,800, 153,000)	25.4 (16.7, 39.4)
Ceará	31,800 (29,000, 35,000)	13.3 (12.1, 14.6)	34,700 (18,400, 57,800)	14.4 (7.7, 24.1)	66,000 (49,300, 88,500)	27.5 (20.5, 36.9)
Distrito Federal	7980 (6940, 9290)	10.8 (9.4, 12.5)	6090 (0, 13,500)	8.2 (0, 18.2)	14,000 (7880, 21,800)	18.9 (10.6, 29.4)
Espírito Santo	12,200 (11,000, 13,500)	12 (10.8, 13.3)	8540 (3160, 16,800)	8.4 (3.1, 16.5)	20,600 (15,100, 29,100)	20.3 (14.9, 28.6)
Goiás	22,800 (20,800, 25,300)	12.4 (11.3, 13.8)	17,500 (6370, 36,800)	9.5 (3.5, 20)	40,100 (28,400, 59,600)	21.8 (15.4, 32.4)
Maranhão	28,500 (25,500, 31,900)	12.7 (11.4, 14.2)	37,100 (21,000, 56,400)	16.5 (9.4, 25.2)	65,200 (48,600, 85,000)	29.1 (21.7, 37.9)
Mato Grosso	13,200 (11,300, 16,500)	13.4 (11.5, 16.8)	9760 (2340, 22,400)	9.9 (2.4, 22.8)	22,800 (14,600, 35,200)	23.2 (14.9, 35.8)
Mato Grosso do Sul	10,300 (9440, 11,300)	13.5 (12.4, 14.7)	8920 (2300, 17,600)	11.7 (3, 23)	19,100 (12,300, 27,700)	25 (16.1, 36.3)
Minas Gerais	52,500 (48,600, 56,300)	10.8 (10, 11.6)	52,200 (21,400, 101,000)	10.7 (4.4, 20.8)	104,000 (71,700, 153,000)	21.4 (14.7, 31.5)
Pará	30,600 (27,600, 34,200)	11.2 (10.1, 12.6)	40,500 (22,000, 66,500)	14.9 (8.1, 24.5)	70,400 (51,700, 95,700)	25.9 (19, 35.2)
Paraíba	15,200 (13,800, 17,100)	14.4 (13.1, 16.2)	15,800 (0, 29,400)	15 (0, 27.9)	30,800 (14,500, 44,600)	29.2 (13.8, 42.3)
Paraná	34,700 (31,600, 37,700)	12.5 (11.4, 13.6)	25,700 (8390, 50,000)	9.2 (3, 18)	60,100 (42,600, 85,100)	21.6 (15.3, 30.6)
Pernambuco	35,300 (31,800, 38,900)	13.7 (12.3, 15.1)	37,000 (18,500, 63,200)	14.3 (7.2, 24.5)	71,900 (53,200, 99,000)	27.8 (20.6, 38.3)
Piauí	11,100 (9900, 12,400)	12.4 (11, 13.8)	9770 (0, 23,300)	10.9 (0, 26)	20,700 (10,000, 34,500)	23.1 (11.2, 38.4)
Rio de Janeiro	55,800 (51,300, 60,400)	14.1 (13, 15.3)	59,400 (27,900, 105,000)	15 (7.1, 26.6)	114,000 (82,800, 160,000)	29 (21, 40.4)
Rio Grande do Norte	12,600 (11,100, 15,100)	13.9 (12.3, 16.8)	12,100 (0, 22,900)	13.4 (0, 25.4)	24,400 (11,700, 36,000)	27.1 (13, 39.9)
Rio Grande do Sul	30,700 (27,900, 33,900)	12.2 (11.1, 13.5)	25,400 (8980, 52,100)	10.1 (3.6, 20.7)	55,700 (39,200, 81,700)	22.1 (15.5, 32.4)
Rondônia	7200 (6200, 8500)	14.4 (12.4, 17)	5430 (0, 11,700)	10.9 (0, 23.5)	12,600 (6990, 19,000)	25.1 (14, 38.1)
Roraima	3500 (3130, 4130)	17.5 (15.6, 20.6)	2810 (945, 5420)	14 (4.7, 27.1)	6260 (4290, 8870)	31.3 (21.4, 44.3)
Santa Catarina	16,000 (14,600, 17,400)	9.5 (8.7, 10.4)	13,700 (3970, 27,800)	8.2 (2.4, 16.6)	29,500 (19,600, 43,900)	17.6 (11.7, 26.2)
São Paulo	133,000 (119,000, 146,000)	12.2 (10.9, 13.5)	107,000 (50,100, 188,000)	9.9 (4.6, 17.2)	239,000 (182,000, 322,000)	22 (16.8, 29.7)
Sergipe	9890 (8900, 11,200)	15.7 (14.1, 17.8)	6780 (0, 13,100)	10.8 (0, 20.7)	16,600 (10,100, 23,200)	26.3 (16, 36.9)
Tocantins	5470 (4850, 6080)	11.8 (10.4, 13.1)	3580 (0, 9840)	7.7 (0, 21.2)	8970 (4850, 15,300)	19.3 (10.4, 33)
Total	673,000 (652,000, 690,000)	12.6 (12.2, 12.9)	635,000 (534,000, 758,000)	11.9 (10, 14.2)	1,300,000 (1,190,000, 1,430,000)	24.3 (22.3, 26.7)

Estimates are reported as central estimates with 95% uncertainty intervals in parentheses. All-cause loss of parents and/or grandparents or older kin includes children who experienced the loss of both a parent and a grandparent or older kin; these children are not double-counted.

In absolute terms, the three worst affected states were São Paulo with a total of 133,000 (119,000–146,000), Rio de Janeiro with a total of 55,800 (51,300–60,400), and Minas Gerais with a total of 52,500 (48,600–56,300) children experiencing all-cause orphanhood. These are the three most populous states of Brazil. When calculating state-level orphan incidence rates per 1000 children, the three worst affected regions were the state of Roraima with 17.5 per 1000 children (15.6–20.6), Sergipe with 15.7 per 1000 (14.1–17.8), and Rondônia with 14.4 per 1000 (12.4–17.0). We additionally present estimates disaggregated by the five macro regions in Supplementary material section 2.5.

Over the same time period, we estimate a total of 635,000 (534,000–758,000) children in Brazil lost at least one co-residing grandparent or older kin (an adult living in the same household aged 60+) due to any cause of death (Table 1, Fig. 1). Of these, 16,200 (3350–46,600) lost multiple co-residing grandparents and/or older kin, and 91,800 (53,700–150,000) lost a grandparent or older kin with direct responsibility (i.e., there were no adults aged 18–59 living in the same household, although there may have been other grandparents). A relatively small number of these children may also have lost a parent—we account for this in Table 1.

In absolute terms, the three worst affected states were São Paulo with a total of 107,000 (50,100–188,000) children losing at least one co-residing grandparent or older kin, Rio de Janeiro with a total of 59,400 (27,900–105,000) children affected, and Minas Gerais with a total of 52,200 (21,400–101,000) affected. Incidence rates of orphanhood per-1000-children due to death of a co-residing grandparent or older kin was worst in the state of Amazonas with 17.1 per 1000 children (9.4, 27.5), Maranhão with 16.5 per 1000 children (9.4–25.2), and Alagoas with 15.4 per 1000 children (0, 29.4). Tables for all categories and stratifications of orphanhood can be found in Supplementary material section 4.

### COVID-19-associated orphanhood

We estimate that, from January 2020 through December 2021, a total of 284,000 (95% uncertainty interval 235,000, 348,000) children in Brazil experienced or grand/or grandparents or older kin (two-year incidence) due to COVID-19-associated causes (two-year incidence).

We estimate that from January 2020 through December 2021, a total of 149,000 (144,000–154,000) children in Brazil experienced COVID-19-associated orphanhood as estimated through excess mortality (Table 2, Fig. 1). Of these, 105,000 (100,000–109,000) were orphaned due to the loss of a father accounting for 70.5% of all orphans, 43,800 (41,600–45,800) were orphaned due to the loss of a mother accounting for 29.4% of all orphans, and 160 (130–200) were orphaned due to the loss of both parents accounting for 0.1% of all orphans. Like all-cause related orphanhood, COVID-19-associated orphanhood was estimated to be greater in older children than younger children (Fig. 2).

**Table 2:** Estimates of orphanhood due to coronavirus disease 2019 (COVID-19)-associated deaths and estimates of the number of children that lost a co-residing grandparent or other older kin.

Federative unit	COVID-19-associated orphanhood		COVID-19-associated loss of grandparents or older kin		COVID-19-associated loss of parent and/or grandparents or older kin	
	Total	Per 1000 children	Total	Per 1000 children	Total	Per 1000 children
Acre	689 (534, 859)	2.2 (1.7, 2.7)	720 (0, 2690)	2.3 (0, 8.6)	1410 (621, 3340)	4.5 (2, 10.7)
Alagoas	3070 (2690, 3520)	3.2 (2.8, 3.6)	2860 (127, 10,000)	2.9 (0.1, 10.3)	5920 (3100, 12,900)	6.1 (3.2, 13.2)
Amapá	892 (719, 1080)	3.1 (2.5, 3.7)	1020 (0, 4190)	3.5 (0, 14.4)	1910 (842, 5050)	6.5 (2.9, 17.3)
Amazonas	5220 (4530, 5900)	3.6 (3.1, 4.1)	8620 (2770, 18,300)	6 (1.9, 12.7)	13,800 (7810, 23,300)	9.6 (5.4, 16.2)
Bahia	10,800 (9830, 12,000)	2.8 (2.5, 3.1)	6030 (0, 30,200)	1.6 (0, 7.8)	16,900 (10,200, 40,900)	4.3 (2.6, 10.5)
Ceará	6970 (6340, 7700)	2.9 (2.6, 3.2)	6430 (895, 18,700)	2.7 (0.4, 7.8)	13,400 (7790, 25,600)	5.6 (3.2, 10.7)
Distrito Federal	1750 (1430, 2180)	2.4 (1.9, 2.9)	2230 (0, 7790)	3 (0, 10.5)	3980 (1690, 9660)	5.4 (2.3, 13)
Espírito Santo	2390 (2080, 2740)	2.4 (2, 2.7)	1950 (0, 6840)	1.9 (0, 6.7)	4340 (2300, 9420)	4.3 (2.3, 9.3)
Goiás	6010 (5370, 6830)	3.3 (2.9, 3.7)	4670 (0, 18,200)	2.5 (0, 9.9)	10,700 (5840, 24,200)	5.8 (3.2, 13.2)
Maranhão	5960 (5210, 6820)	2.7 (2.3, 3)	6980 (1580, 17,500)	3.1 (0.7, 7.8)	12,900 (7440, 23,600)	5.8 (3.3, 10.5)
Mato Grosso	4350 (3780, 5230)	4.4 (3.9, 5.3)	3060 (0, 12,900)	3.1 (0, 13.2)	7400 (4090, 17,000)	7.5 (4.2, 17.3)
Mato Grosso do Sul	2930 (2600, 3290)	3.8 (3.4, 4.3)	2070 (0, 7930)	2.7 (0, 10.4)	4990 (2870, 10,700)	6.5 (3.8, 14)
Minas Gerais	10,200 (9210, 11,100)	2.1 (1.9, 2.3)	9510 (60, 36,300)	2 (0, 7.4)	19,700 (10,400, 46,300)	4 (2.1, 9.5)
Pará	3930 (3210, 4820)	1.4 (1.2, 1.8)	9110 (1850, 23,800)	3.4 (0.7, 8.8)	13,000 (5660, 28,700)	4.8 (2.1, 10.5)
Paraíba	3500 (3070, 3970)	3.3 (2.9, 3.8)	2910 (78, 10,400)	2.8 (0.1, 9.9)	6400 (3490, 13,900)	6.1 (3.3, 13.2)
Paraná	10,200 (9280, 11,100)	3.7 (3.3, 4)	6260 (165, 21,500)	2.3 (0.1, 7.7)	16,400 (10,300, 31,700)	5.9 (3.7, 11.4)
Pernambuco	6880 (6100, 7710)	2.7 (2.4, 3)	6480 (503, 20,100)	2.5 (0.2, 7.8)	13,400 (7260, 26,900)	5.2 (2.8, 10.4)
Piauí	2170 (1830, 2510)	2.4 (2, 2.8)	2730 (0, 10,900)	3 (0, 12.2)	4900 (2190, 12,800)	5.5 (2.4, 14.3)
Rio de Janeiro	12,900 (11,600, 14,100)	3.3 (2.9, 3.6)	11,800 (1030, 34,700)	3 (0.3, 8.8)	24,600 (13,600, 47,600)	6.2 (3.5, 12.1)
Rio Grande do Norte	1800 (1500, 2160)	2 (1.7, 2.4)	1910 (0, 7270)	2.1 (0, 8.1)	3710 (1740, 8990)	4.1 (1.9, 10)
Rio Grande do Sul	5900 (5180, 6670)	2.3 (2.1, 2.6)	4570 (0, 18,800)	1.8 (0, 7.5)	10,500 (5580, 24,600)	4.1 (2.2, 9.8)
Rondônia	2160 (1800, 2630)	4.3 (3.6, 5.3)	2040 (201, 6700)	4.1 (0.4, 13.4)	4190 (2240, 8830)	8.4 (4.5, 17.7)
Roraima	745 (566, 924)	3.7 (2.8, 4.6)	692 (55, 2230)	3.5 (0.3, 11.1)	1430 (741, 2970)	7.2 (3.7, 14.8)
Santa Catarina	2720 (2400, 3110)	1.6 (1.4, 1.9)	3430 (0, 13,300)	2.1 (0, 7.9)	6140 (2670, 16,100)	3.7 (1.6, 9.6)
São Paulo	32,100 (27,700, 35,600)	3 (2.5, 3.3)	24,700 (3180, 68,000)	2.3 (0.3, 6.3)	56,700 (34,500, 101,000)	5.2 (3.2, 9.3)
Sergipe	1630 (1350, 1940)	2.6 (2.1, 3.1)	1200 (0, 5410)	1.9 (0, 8.6)	2830 (1520, 7180)	4.5 (2.4, 11.4)
Tocantins	1270 (1070, 1480)	2.7 (2.3, 3.2)	1460 (0, 5940)	3.1 (0, 12.8)	2730 (1200, 7150)	5.9 (2.6, 15.4)
Total	149,000 (144,000, 154,000)	2.8 (2.7, 2.9)	135,000 (85,900, 199,000)	2.5 (1.6, 3.7)	284,000 (235,000, 348,000)	5.3 (4.4, 6.5)

Estimates are reported as central estimates with 95% uncertainty intervals in parentheses. All-cause loss of parents and/or grandparents or older kin includes children who experienced the loss of both a parent and a grandparent or older kin; these children are not double-counted.

In absolute terms, the three worst affected states were São Paulo with a total of 32,100 (27,700–35,600), Rio de Janeiro with a total of 12,900 (11,600–14,100), and Bahia with a total of 10,800 (9830–12,000) children experiencing COVID-19 orphanhood. Excluding Minas Gerais (the second most populous state), these are the three most populous states in Brazil. However, on a per-1000-children basis, the three worst affected states were Mato Grosso with 4.4 (3.9–5.3) orphans per 1000 children, Rondônia with 4.3 (3.6–5.3) orphans per 1000 children, and Mato Grosso do Sul with 3.8 (3.4–4.3) orphans per 1000 children.

**Fig. 3:**
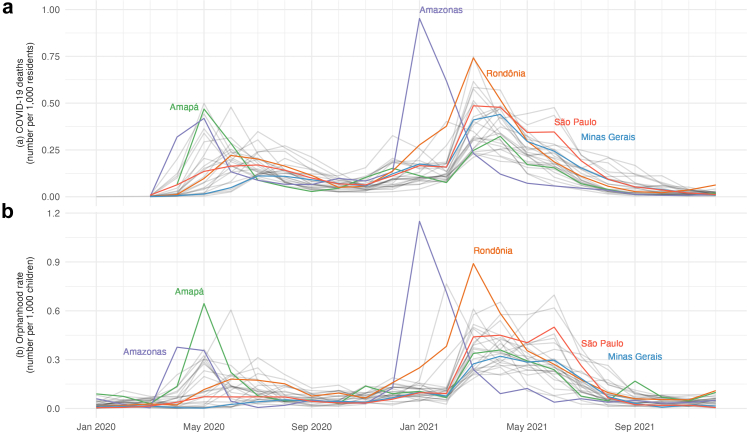
COVID-19 reported deaths (a) by state for all ages per 1000 residents.^25^ Monthly estimates of the number of children (b) ages 0 to 17 years-old experiencing COVID-19 associated orphanhood (loss of one or both parents due to excess mortality) in Brazil from January 2020 through December 2021 per 1000 children. Note that while there is a striking correspondence between the two sets of time series, our estimates of orphanhood in (b) were not derived from (a).

Fig. 3 presents monthly estimates of the number of children experiencing COVID-19-associated orphanhood and shows that the state of São Paulo (the state with the greatest number of children, 10.8 m), had an orphanhood rate approximately half that of Minas Gerais (the state with the next highest number of children, 4.9 m), resulting in the two states experiencing comparable levels of orphanhood in absolute terms. Only between March 2021 and July 2021 was orphanhood in absolute terms significantly greater in the state of São Paulo.

Over the same time period, we estimate a total of 135,000 (85,900–199,000) children in Brazil lost at least one co-residing grandparent or older kin (an adult living in the same household aged 60+) due to COVID-19-associated mortality (Table 2, Fig. 1). Of these, 540 (0–4130) lost multiple co-residing grandparents and/or older kin, and 21,300 (5870–62,900) lost a grandparent or older kin with direct responsibility (i.e., there were no adults aged 18–59 living in the same household, although there may have been other grandparents).

In absolute terms, the three worst affected states were São Paulo with a total of 24,700 (3180–68,000) children losing at least one co-residing grandparent or older kin, Rio de Janeiro with a total of 11,800 (1030–34,700) children affected, and Minas Gerais with a total of 9510 (60–36,300) affected. Incidence rates of orphanhood per-1000-children due to death of a co-residing grandparent or older kin was worst in the state of Amazonas with 6.0 per 1000 children (1.9–12.7), Rondônia with 4.1 per 1000 children (0.4–13.4), and Amapá with 3.5 per 1000 children (0–14.4).

### Comparisons with administrative data

#### Arpen-Brasil Civil Registry data

Of the 12,211 COVID-19 deaths of children aged 6 or under, the sex of the parent was recorded as male in 8145 (66.7%) of cases and female in 3960 (32.4%) of cases. Double orphans (loss of both parents) accounted for 103 (0.8%) children. These proportions are similar to our estimates of 70.5% paternal versus 29.4% maternal orphans for COVID-19 associated orphans. We estimated the loss of both parents to account for only 0.1% of orphaned children, a lower percentage than in the Arpen-Brasil dataset, but we note that we were unable to verify whether double orphans could have been double counted in some cases in the Arpen-Brasil dataset.

**Fig. 4:**
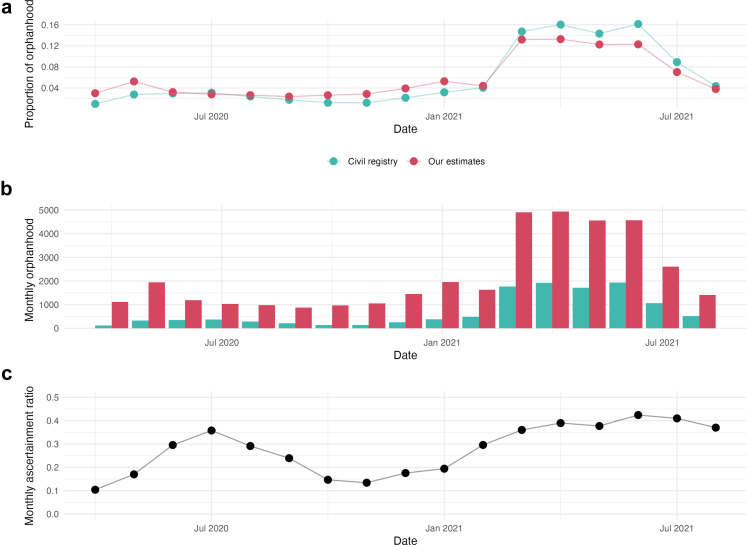
The proportion of COVID-19-associated orphanhood in 6-year-olds and under that occurred from April 2020 through August 2021 by month and source of data (panel a), monthly orphanhood (panel b), and monthly ascertainment ratio of civil registry data relative to our estimates (panel c).

The Arpen-Brasil data are reported by date-of-death from 18 March 2020 through 24 September 2021. Of these, 12,010 (98.4%) were reported from 1 April 2020 through 31 August 2021. We calculated the proportion of total orphanhood over this latter period that occurred by month for the civil registry data and our estimates (Fig. 4) and found similar proportions over time.

However, the magnitude of our model estimates differed from the registry data. Using our monthly COVID-19-associated orphanhood methods and adjusting so the total matches the central estimates from the full model, we estimate 37,200 children aged 6 and under experienced orphanhood from 1 April 2020 through 31 August 2021. Dividing the 12,010 from the Arpen-Brasil dataset by 37,200 yields a civil registry ascertainment rate of 32.3%. We further estimate that, over this period, 27,100 children aged 6 and under lost a father and 10,100 children aged 6 and under lost a mother using our methods. The civil registry data estimates 8079 children as having lost a father and 3928 as having lost a mother, corresponding to ascertainment rates of 29.8% (for paternal orphanhood) and 38.9% (for maternal orphanhood). Ascertainment also varied by month, with lower values in early-2020 (10.4% in April 2020, the month with lowest ascertainment) and late-2020 (13.4% in November 2020), and higher values in mid-2020 (35.8% in July 2020) and 2021 (42.4% in June 2021, the month with the greatest ascertainment).

#### Campinas data

Manual review of death certificates in Campinas found that from 481 children, 434 children lost a mother or father, 1 lost a grandfather, and 46 lost an unknown caregiver from COVID-19. Of the 434 that lost a mother or father, 293 (67.5%) were listed as having lost a father, and 141 (32.5%) were listed as having lost a mother. These are similar proportions of paternal versus maternal orphans to our estimates and to those from Arpen-Brasil. These data also support our conclusions that orphanhood occurs more in older children (Supplementary material section 1.2.4).

Campinas, population 1.14 m, accounts for 2.5% of São Paulo State and 2.4% of children aged 0 to 19.^26^ Multiplying our estimates of orphanhood in São Paulo State by 2.4% yields 543 paternal orphans, 233 maternal orphans, and 1 double orphan. This corresponds to an ascertainment rate of 54% for paternal orphans, 61% for maternal orphans, and 56% overall.

## Discussion

By leveraging new data sources, in Brazil between January 2020 and December 2021, we estimated that 673,000 children (more than 1 in 100 total) experienced orphanhood from all-causes, and a total of 1.3 million children experienced death of parents, co-residing grandparents, or older kin. Our estimates of maternal orphanhood due to COVID-19 associated causes are similar to those of,^16^ who estimated that a total of 40,830 children in Brazil lost their mother due to COVID-19. Orphanhood and caregiver loss are a permanent loss, and although alternative care arrangements have been found to alleviate negative outcomes, provision and careful planning is needed in both the short and longer term. During the pandemic, social support and bereavement processes were disrupted. School closures, health care diversion, and social isolation severely interrupted essential pathways for care, support and attention. Cultural mourning and bereavement experiences were also restricted by physical distancing measures as was the ability to seek help.

In planning for future epidemics, it is important to note that COVID-19-associated causes are a large contributing factor to all-cause orphanhood and caregiver loss among children in Brazil. Our state specific estimates highlight substantial differences with the most affected state (Mato Grosso, 4.4 children per 1000 estimated to have lost one or both parents) having an estimated rate of orphanhood 3.1 times that of the least affected state (Pará, 1.4 per 1000). Brazil, the most populous and largest country in Latin America, is also socio-economically (e.g., housing and employment) and geographically (e.g., access to services) one of the most unequal.^27^ Prior studies looking at COVID-19 mortality have shown that socio-economic vulnerabilities were major drivers in mortality outcomes during the period of the pandemic,^14^ beyond population age structure and prevalence of chronic diseases. Our findings from a geographic perspective are consistent with the findings of that prior research and highlight similar areas where socio-economic vulnerabilities are more salient, primarily in the Northern regions.^14^^,^^28^ Our research further expands prior findings in that it addresses all-cause mortality among caregivers and its differential impact on orphanhood.

Many causes of orphanhood beyond COVID-19 are important to address from a prevention point of view. While the effects of that pandemic have largely subsided, other causes are ongoing. One such cause is violence in its multiple expressions which affects mostly younger populations many of which are likely to have younger children in their families Interpersonal violence constitutes the top cause of death for women aged 30–34 in Brazil^29^ with intimate partner violence potentially being an important cause of orphanhood for children bereft of mothers in this age range. In its most lethal manifestation, Brazil ranks first in terms of counts of homicide deaths globally and has rates over 20 per 100,000 population in the American continent.^30^ The Americas report the highest homicide rates per 100,000 population globally, mainly affecting younger males (ages 15-29 years-old).^30^ While in recent years, national trends show reductions in homicide, subnationally, trends are very different at State levels.^31^

While attention has been drawn to COVID-19 orphans in research publications^6^^,^^19^ and policy briefs,^11^ especially in Brazil,^32^ they have to date been based on modelling approaches, combining epidemiological, demographic, and statistical methods and at the national level only. While these methods are especially valuable in data poor settings, wherever possible these approaches should be augmented by analyses of directly-observed data, such as administrative or survey data. With the notable exception of Brazil, these data simply did not exist in the early years of the pandemic (and we are unaware of any comprehensive efforts subsequently). The situation in Brazil thus presented a unique opportunity to partially validate the modeling methods and to characterize the degree to which administrative data undercounts orphans.

When comparing the fraction of maternal and paternal orphans in the Arpen-Brasil Civil Registry dataset and the Campinas dataset to our modeled estimates, there was a very close match. The same was true of a temporal match between our modeled estimates and the Arpen-Brasil dataset. However, there was a stark difference in the magnitude of the number of children affected. Arpen-Brasil’s numbers were about one third as high as our estimates, and the Campinas numbers about one half, both of which are to be expected with administrative data. There are two reasons why administrative data would provide an undercount of COVID-19 orphans. First, COVID-19 was itself undercounted in death records in the context of the unfolding pandemic in 2020–21.^21^ Secondly, both the Arpen-Brasil and Campinas datasets that we used relied on death certificates. While Brazil is unique in the world in that death certificates record information on child dependents of the deceased,^12^ this field is not digitally available and requires local work manually checking each certificate, and we would never expect this type of administrative data collection to yield complete data. Similarly, the Arpen-Brasil dataset relied on a matching process carried out by individual Civil Registry offices across Brazil between death and birth certificates (a process that was on the whole only possible for children aged 0 to 6 due to recent changes in how identification numbers are now assigned at birth). This type of matching with governmental records is never perfect, and birth certificates may fail to list fathers.^33^ Implied ascertainment in the Civil Registry data (Fig. 4c) also varied substantially over time, with changes occurring in a smooth manner rather than through abrupt fluctuations. This pattern suggests the presence of a genuine underlying signal rather than random noise. The factors driving these variations should be investigated to enhance our understanding.

From a legislative perspective Brazil also addresses orphanhood linked to violence specifically due to feminicide. Law No. 14,717/2023, establishes a special pension for children and dependents orphaned due to feminicide. This problem affects mostly younger women and hence has a great impact on children who are left without caregivers. The law focuses on the protection and support of indirect victims of gender-based violence.

Our methods also have limitations. We use excess mortality to approximate COVID-19-associated deaths to eliminate bias associated with cause-of-death reporting, although our estimates potentially include non-COVID-related deaths (biasing orphanhood estimates upwards) or undercount COVID-related deaths if societal interventions resulted in a reduction in other causes of mortality (biasing orphanhood estimates downwards).^3^ Various simplifying probabilistic assumptions were made, such as the independence between the probability that both parents die, when co-residing family members share many exposures such as to infectious pathogens or environmental hazards. The independence assumption likely results in us undercounting double orphanhood, and thus slightly overcounting total orphanhood. We also assume that fertility and mortality are independent. If parents with more children were more likely to die, then we will underestimate the true rate of orphanhood (and vice-versa). These data were also not available disaggregated by ethnicity so no conclusions could be drawn about this.

While the PNS survey (used for male fertility and household structure estimates) features a large sample size, there are still high levels of uncertainty when estimating quantities at the state-level or by the age-of-child. Multiple approximations were required when using other data sources also, such as the interpolation of population count data prior to 2010, and the application of point estimates of live births underreporting from a single year to multiple years of births data. These approximations are small and are expected to cause only small bias, but the extent of this bias is again difficult to quantify. We are also limited by the timeframe covered by the data we used, while excess mortality estimates covered January 2020 through December 2021, the COVID-19 public health emergency continued until May 2023,^34^ meaning additional COVID-19-associated orphanhood will have occurred after the conclusion of our study period. We also lacked the data required to perform uncertainty quantification on a monthly basis, thus preventing the separation of the early 2020 pre-pandemic period from later months, however, low excess mortality during that time suggests minimal impact on our estimates.

Given both the high magnitude and concerning life-long consequences of orphanhood from all causes in Brazil, sources of administrative data like those cited in this report could be used to identify children affected by orphanhood in real time and link them to care. Specifically, further research should evaluate the effectiveness of using these sources of orphanhood data to refer surviving caregivers to evidence-based parenting, economic, and educational support, to ensure these children are protected from negative orphanhood-linked outcomes. These data are also useful in identifying how health issues differentially affect populations by geography and socio-economic status. Consequently, it can contribute to a more effective and egalitarian allocation of resources for prevention. Where administrative data are not available, future research should seek to incorporate additional sources of data to produce higher-quality estimates of orphanhood. Efforts to protect orphans due to all causes may be further improved through legislation that integrates different causes of mortality among caregivers and links it to protection measures for children and dependents. Brazil is in a unique position to lead globally on this front as it has already a robust infrastructure of data that if improved can further enhance the country’s longstanding public health focus on primary prevention.

### Conclusion

The COVID-19 pandemic serves as an example of government reaction and preparedness for a crisis. The sharp, rapid, and comprehensive response needed for children can only begin to be effective if it is based on accurate, targeted, and available data to guide the response. This study has important policy implications for governments who continue to struggle with how to effectively design and deliver social assistance to orphans. The first step in policy is the accurate identification of the target population in need. Official death certificates are effective in helping to identify orphans, but administrative data alone are insufficient. In Brazil in particular, the evidence presented in this study suggests that there could be sizable shares of orphans in the groups of children 7 years and older, including adolescents, who are wholly missing from the nationwide administrative data. As we noted earlier, despite the magnitude of the number of caregivers lost to COVID-19, a national program to monitor and provide a broad array of services to orphans does not exist in Brazil. Most of the legislation introduced in 2021 is still under discussion in the Senate. This provision may serve as a global blueprint for child crisis provision. Addressing the limitations of information systems aimed at identifying and monitoring these children at risk may increase the accuracy of estimates and better inform public health responses. Urgent attention is warranted to refine these systems so that the response to the next crisis meets the needs of so many children who currently fall under the radar.

## Contributors

Conceptualisation: NS, HJTU, ASB, LC, SH, MCC, SF. Methodology: NS, HJTU, AB, ASB, OR, JP, MCC, SF. Investigation: NS HJTU, JP, AV, LM, LC, SH, ASS, MCC, SF, RPS. Software: NS. Data curation: NS, HJTU, JP, ES, AVRA, RPS, ASS, MCC, SF. Visualisation: NS, HJTU, SF. Validation: NS, HJTU, SF. Writing—original draft: NS, HJTU, AV, LC, SH, LB, MCC, SF. Writing—review and editing: NS, HJTU, AV, LS, AVRA, SH, LR, LB, MCC, SF. Decision to submit: SF.

## Data sharing statement

All code and data (where we are able to provide directly) required to reproduce these results are available at https://github.com/MLGlobalHealth/BrazilOrphanhood. Where we are unable to provide original data, instructions are provided in this repository on how to obtain the data. A flowchart of the structure of the code is provided in Supplementary Fig. S5.

## Ethics and use of AI

The study used only open available data. Death certificates in Brazil are public documents (federal law n. 6015 issued on December 31, 1973) and were obtained and reviewed for Campinas by co-author Andrea Santos Souza. Administrative data on COVID-19 orphans were shared by the Associação Nacional dos Registradores de Pessoas Naturais (Arpen-Brasil).

Generative AI technology (ChatGPT) was used to support code development, primarily for the creation of figures. No AI-generated code was directly used in producing the final results.

## Editor note

The Lancet Group takes a neutral position with respect to territorial claims in published maps and institutional affiliations.

## Declaration of interests

No authors have conflicting interests. Details of grant funding are provided in the acknowledgements. HJTU and OR also declare funding from Moderna charitable foundation for a different project about orphanhood.

## Appendix A Supplementary data

Supplementary materials

Translated abstract
