# Supplementary material for: Regional and national estimates of children affected by all-cause and COVID-19-associated orphanhood and caregiver death in Brazil, by age and family circumstance: a modeling study
Source: Lancet Reg Health Am. 2025 Sep 29;51:101252. doi: 10.1016/j.lana.2025.101252 (PMC12513060; doi:10.1016/j.lana.2025.101252)
Supplement: Supplementary materials [file mmc1.pdf]

Regional and national minimum estimates of children affected by COVID-19-associated orphanhood and caregiver death in Brazil, by age and family circumstance - Supplementary Material

Contents

1 Supplementary Methods **2**
- 1.1 Data . . . 2
  - 1.1.1 All-cause mortality . . . 2
  - 1.1.2 Excess mortality . . . 2
  - 1.1.3 Live births . . . 2
  - 1.1.4 Under-reporting of live births . . . 2
  - 1.1.5 Child mortality . . . 3
  - 1.1.6 PNS 2019 . . . 3
  - 1.1.7 Population estimates . . . 4
  - 1.1.8 Administrative data . . . 4
- 1.2 Methods . . . 4
  - 1.2.1 Estimating parental orphanhood . . . 4
  - 1.2.2 Estimating the number of children born to women . . . 5
  - 1.2.3 Estimating the number of children born to men . . . 6
  - 1.2.4 Estimating double orphanhood . . . 8
  - 1.2.5 Estimating the loss of co-residing grandparents and older kin . . . 9
  - 1.2.6 Estimating the number of children experiencing either parental orphanhood and the loss of co-residing grandparents and older kin . . . 10
  - 1.2.7 Estimating monthly orphanhood . . . 10
  - 1.2.8 Structure of code . . . 11

2 Supplementary results **12**
- 2.1 Children experiencing both parental orphanhood and the loss of co-residing grandparents and older kin . . . 12
- 2.2 Regional orphanhood incidence by child age . . . 12
- 2.3 Annual age-specific male fertility rates . . . 12
- 2.4 Age-distribution of children listed in the Campinas administrative data . . . 12
- 2.5 Orphanhood estimates by region . . . 13

3 Supplementary figures **14**

4 Supplementary tables **22**

1 Supplementary Methods

1.1 Data

1.1.1 All-cause mortality

All-cause mortality data were obtained by federative unit, 10-year-age-group, sex, and month for 2020 and 2021 from [1]. We present these data, aggregated by federative unit and year, and standardised by population count (Supplementary Section 1.1.7) in Supplementary Figure 1.

1.1.2 Excess mortality

Central estimates and 1,000 simulated samples of excess mortality by federative unit, 10-year-age-group, and sex for 2020 and 2021 were obtained from [1]. We present these data, aggregated by federative unit and year, and standardised by the population data later described in Supplementary Section 1.1.7 in Supplementary Figure 2. Prior to aggregating these data, we set any observations of negative excess mortality to 0.

Central estimates of excess mortality further disaggregated by month (in addition to federative unit, age-group, and sex) were also obtained from the same source, although uncertainty (in the form of samples from a posterior distribution) were unavailable by month.

1.1.3 Live births

We obtained live births line-data from Sistema de Informações sobre Nascidos Vivos [2] and used it to estimate female fertility (Supplementary Section 1.2.2). These data consist of one line per birth. Prior to 2010, the date, mother's age at the time of birth, and state of residence were recorded. From 2010 onwards, the dataset additionally included the father's age at the time of birth, although a large proportion of records are missing these data (Supplementary Section 2.3).

Live births line-data are processed by grouping by the year, federative unit, and age-of-parent, and then summing the number of rows in each group. Supplementary Figure 3 reports the annual number of live births recorded by the age of the mother and, where available, the age of the father. A small proportion of births are also missing the age of the mother (no more than 1.3% in any given year and federative unit) - we do not account for this in Supplementary Figure 3, but we do when calculating fertility rates (Supplementary Section 1.2.2).

The initial processing of these data is performed by scripts in the 'data/population/' folder of the GitHub repository. This is also the location where the processed data are stored.

1.1.4 Under-reporting of live births

Estimates of under-reporting of live births for 2020 were obtained from [3]. These were estimated using the capture-recapture technique and are reported at the national level for women aged between 15 and 49. Overall under-reporting rates are estimated to be low: ranging from 0.52% in women aged 38 years to 5.32% in women aged 49 years. Only women aged 45 and above had under-reporting rates above 1%, a group that accounts for a small proportion of total births. We

describe how these estimates are used to account for under-reporting in Supplementary Section 1.2.2.

1.1.5 Child mortality

As we leverage live births data when calculating the mean number of children that women currently have, we must account for child mortality. Life tables for the population of Brazil were obtained by single-age and year (between 2003 and 2020) from [4] containing $q_{a,y}$, the probability that an individual of age a survives until age $a+1$ in year y. These are used in Supplementary Section 1.2.2 to calculate the probability that a child survives until reaching (at least) age c in year y.

1.1.6 PNS 2019

The National Health Survey (PNS) 2019 [5] is a survey representative of Brazil's population that reside in private households at the state level. The survey consists of 279,382 observations from 94,114 households. The questionnaire is divided into three sections:

(i) the household questionnaire,

(ii) a questionnaire for all residents in the household, and

(iii) a questionnaire for a selected resident.

Sections (i) and (ii) consist of a comparatively limited number of questions, whereas section (iii) consists of more detailed questions and is completed by a randomly selected individual aged 15+ from the household.

The sampling scheme consists of three stages:

(a) primary sampling unit stratification using census tracts with each tract chosen proportional to the number of private dwellings,

(b) the selection of households within each primary sampling unit by simple random sampling, and

(c) the selection at random of a single resident aged 15 years old or over for section (iii).

A section on paternity and male-partner prenatal care was introduced in the 2019 survey which asked questions related to fatherhood. This section was included in the survey when the randomly selected respondent in stage (c) was male. Specifically the respondent was asked whether they have had a biological child, the number of male and female children, the age of their youngest child, and the year-of-birth of their oldest child. This almost allows for the direct calculation of the number of children currently aged ≤ 17 years-old, with minor assumptions required when there are at least three children, the youngest child is < 18 years-old, and the oldest child is ≥ 18 years-old.

PNS 2019 microdata is publicly available and can be retrieved from the IBGE website. An R package, PNSIBGE, provides functions that download and analyse the PNS [6].

1.1.7 Population estimates

Two sources of population estimates were used.

"Population projections by simple sex and age" from 2018, updated on 12/09/2020 [7], provides population estimates by sex, federative unit, and single-year age for 2010 onwards. We use these data when current population counts (assumed to be the population in 2020) are required.

Prior to 2010, population estimates by sex and federative unit are disaggregated in 5-year age-groups only. These files are titled "Population projections by sex and age - updated on April 6, 2020" from the 2018 publication, which we use for 2010 onwards, and "Population projection by sex and age" from the 2013 publication, which we use for estimates prior to 2010 [7]. We use these data when historical population counts are required. Supplementary Figure 4 presents these data aggregated into 10-year age-groups.

1.1.8 Administrative data

We compare our results to two sources of administrative data on COVID-19-related orphanhood in Brazil.

First, Brazil includes a field on death certificates that records child dependents in the household [8]. All death certificates listing COVID-19 as the cause of death in Campinas, a municipality of São Paulo state, were manually reviewed by co-author Andrea Santos Souza and her team. A total of 481 children were identified as having lost a parent to COVID-19.

Second, Brazil's Civil Registry used administrative records to link birth and death certificates, identifying a total of 13,815 orphaned children aged 6-years and under, for the period between 18 March 2020 and 24 September 2021. Of these, 13,431 were listed as orphaned due to COVID-19 deaths, and a further 384 were listed as orphaned due to Severe Acute Respiratory Syndrome (SRAG in Portugese).

1.2 Methods

1.2.1 Estimating parental orphanhood

In order to characterise uncertainty we obtain 1,000 random samples of (excess or all-cause[1]) mortality by age, sex, and state and 1,000 random distributions of age-sex-state specific fertility, where each fertility distribution represents the number of children born to a parent of a given sex and age in a specific state. A single sample of mortality is an integer number of deaths for a given age, sex, and state. A single sample of the fertility distribution for a given age, sex, and state is itself a distribution, from which we can obtain realizations consisting of an integer number of children who might be born to a parent of a given sex and age in a specific state. For brevity, we use "state" to refer to "federative unit" in this section.

Let $O^{(i)}_{a,s,r}$ denote the ith sample of orphanhood resulting from the deaths of parents in age-group a, sex s, and state r in 2020 and 2021. We calculate this quantity using random samples from

[1] All-cause mortality is directly observed, so a samples-based approach is not required. To fit within the wider methodology, we use 1000 identical replicates of observed all-cause mortality.

mortality $E_{a,s,r}$ (Supplementary Section 1.1.2) and fertility $C_{a,s,r}$ as follows:

$$O^{(i)}_{a,s,r} = \sum_{j=1}^{E^{(i_{em})}_{a,s,r}} C^{(i_f,j)}_{a,s,r}$$

where $i_{em} \sim \text{Unif}(\{1,2,\dots,1000\})$, $i_f \sim \text{Unif}(\{1,2,\dots,1000\})$, and we obtain $C^{(i_f,j)}_{a,s,r}$ as iid realisations from the fertility distributions for $j = 1,\dots,E^{(i_{em})}_{a,s,r}$ in a given age-group a/sex s/state r.

By drawing fixed indices i_{em} and i_f for each sample of orphanhood and using these for all combinations of a, s, and r, we ensure that correlations between groups are maintained, thus correctly propagating uncertainty. We describe how fertility estimates $C^{(\cdot,\cdot)}_{a,s,r}$ are generated below.

Central estimates are calculated by taking the mean of the resulting samples, and uncertainty intervals by taking the 2.5 and 97.5 quantiles. While we use a generally Bayesian approach, some components are not Bayesian, notably we utilise survey weights in a frequentist framework. We thus refrain from calling our uncertainty intervals credible intervals, despite them having a similar interpretation.

1.2.2 Estimating the number of children born to women

Annual age-specific female fertility rates were calculated by dividing total live births $B_{a,r,y}$ to women of age a in state r and year y by the corresponding population $N_{a,r,y}$ of women (see Supplementary Sections 1.1.3) and 1.1.7). In groups where no births were recorded, the fertility rate is assumed to be 0. Historical population data by state are only available in 5-year age-groups, so we use the Sprague interpolation method (which preserves the original group totals) to disaggregate this into single-year age-groups [9, 10].

Live births are known to be under-reported [3]. Specifically, there are two sources of under-reporting that we are concerned with: (1) births that are not recorded (Supplementary Section 1.1.4), and (2) live births line-data entries where the mother's age was not recorded (Supplementary Section 1.1.3).

While we know how many entries do not have the mother's age recorded, there is uncertainty associated with the capture-recapture method used to estimate under-reporting. This uncertainty is not provided in the source of data [3]. Furthermore, these estimates are reported nationally for 2020 only, so there is additional uncertainty associated with applying them to other years and by state. To this extent, we allow for considerable uncertainty by modelling under-reporting using a Uniform distribution on $(0, 2p_a)$, where p_a is the estimate of live-births under-reporting in mothers of age a in 2020. For example, a value of $p_a = 0.01$ means 1% of births are not reported. Overall, since p_a is generally small, the introduction of uncertainty in this way has little impact on our results. (Note: estimates of p_a were provided for women aged 15-49, so we apply the average over all age-groups of 0.97% to women aged 50+. Given low fertility rates for women aged 50+, we expect this to have a negligible impact.)

Letting $r_{r,y}$ be the proportion of line-data entries with missing ages for the mother in state r and

year y, we generate samples of annual age-structured female fertility rates using the following procedure:

1. Sample $u \sim \text{Uniform}(0, 1)$
2. For each a, r, and y, set: $f^{(i)}_{a,r,y} = \frac{B_{a,r,y}}{N_{a,r,y}} \frac{(1+2up_a)}{(1-r_{r,y})}$
3. Repeat steps (1-2) for $i = 1, \dots, N$

The resulting $f^{(i)}_{a,r,y}$ is the ith sample of the annual age-specific fertility rate in females of age a in state r, year y.

As we use live births data to estimate total children alive today, we also need to adjust for child mortality. Letting $q_{a,y}$ be the probability that an individual of age a dies before reaching age $a + 1$ in year y as obtained in Supplementary Section 1.1.5, we calculate the probability that a child survives until reaching age c in 2020, denoted q^{survives}_c, using:

$$q^{\text{survives}}_c = 1 - \prod_{a=0}^{c-1} (1 - q_{a,2020-c+a})$$

Thus the ith sample of the mean number of children of age c that a woman of age a in state r has is given by:

$$\bar{C}^{(i)}_{a,\text{female},r,c} = q^{\text{survives}}_c f^{(i)}_{a-c,r,2020-c}$$

These are aggregated into our standard 10-year age-groups A by taking a population-weighted average of the mean number of children born to a woman in each single-year age-group:

$$\bar{C}^{(i)}_{A,\text{female},r,c} = \frac{\sum_{a \in A} N_{a,r} \bar{C}^{(i)}_{a,\text{female},r,c}}{\sum_{a \in A} N_{a,r}}$$

Finally, we assume that the number of living children that a mother has is Poisson-distributed about this mean. The jth realisation of female fertility from the ith fertility distribution is thus generated by sampling a Poisson random variable as:

$$C^{(i,j)}_{A,\text{female},r,c} \sim \text{Poisson}\left(\bar{C}^{(i)}_{A,\text{female},r,c}\right)$$

Samples of the total number of children that a mother has are found by summing over the age-of-child c.

1.2.3 Estimating the number of children born to men

Of the 94,114 individuals that responded to section (iii) of PNS 2019, 44,752 were male and aged 15+. Of these there were 1,953 NA responses, suggesting these individuals were not asked the question, and 126 responses stating “don’t know”. These observations were removed prior to the analysis, leaving a sample size of 42,673. There were no individuals listed as ignoring the question.

If the individual reports having zero, one, or two children we can calculate exactly the number of children aged ≤ 18 at the time of the survey. We can also calculate this quantity exactly if the oldest child is ≤ 18 years-old, or the youngest child is > 18 years-old. There are 3,408 (8.0%) observations for which none of these conditions hold. In this case we randomly assign ages to the middle children uniformly between the age of the youngest and the age of the oldest (inclusive) and use these to calculate the number of children aged ≤ 18. While the uniform assumption may overstate the likelihood of twins and triplets, for example, we do not expect any error induced by this to be substantial.

The *PNSIBGE* [6] and *survey* [11] packages in R were used to download the data (Supplementary Section 1.1.6) and construct a sampling distribution for the number of children born to a male of age-group a in state r. Specifically, for each set of randomly assigned ages to uncertain children i (as in the above paragraph), we create a survey design object using the *pns_design()* function, subset by fathers in age-group a and state r, extract sampling weights using the *weights()* function, and sample the number of children a possible father has $C^{(i,j)}_{a,male,r}$ according to these weights for $j = 1, \dots, n_{eff}$. The effective sample size here is calculated as $n_{eff} = \frac{(\sum w_k)^2}{\sum w_k^2}$ with summation over the weights of fathers in the relevant age-group and state combination. n_{eff} ranges from 25 (in 10-19 year-olds in Goiás) to 401 (in 30-39 year-olds in Maranhão).

When estimating fertility rates by the age-of-child, each sample of $C^{(i,j)}_{a,male,r,c}$ (with an additional subscript c for the age-of-child) for given sampled father is equal to the number of children aged c they are listed as having. Child mortality is accounted for by sampling from a binomial distribution with n equal to the number of children the father has, and p equal to the probability a child survives until age c (Supplementary Section 1.1.5). When estimating total fertility rates, each sample of $C^{(i,j)}_{a,male,r}$ for a given sampled father is equal to the total number of children they are listed as having. Child mortality is accounted for by sampling fro a binomial distribution with n equal to the number of children and p equal to the state-population-weighted-average of child mortality.

If we approximate the age of the father in year y by $a - (2019 - y)$, where a is the age of the father today, and approximate the year each child was born by $2019 - c$, where c is the age of the child today, we can also estimate age-specific fertility rates by year. See Supplementary Section 2.3 for a comparison with live births data.

Potential bias in survey responses

Our samples of the number of children born to males rely upon responses from the randomly selected primary respondent in each household. This means a father living in a large household with many residents aged 15+ is less likely to be selected than a father living in a smaller household. That is, if a father has many children aged 15 to 17 years-old, the survey may be selecting these individuals instead of the father. This introduces a bias: fathers with many older children are less likely to be sampled than those with fewer.

Out of 77,131 households with a possible father (temporarily defined here as a male aged 18+), there are 1,894 (2.5%) instances where a child aged 15-17 was selected as the primary respondent. On average, households containing a possible father where a child aged 15-17 was selected as the

primary respondent contained an average of 2.1 children, compared to 0.71 children in households containing possible fathers where a child was not selected as the primary respondent. As a total of 44,752 possible fathers were selected as the primary respondent, the average number of children per selected possible father could increase from 0.71 to a maximum of 0.76 (a factor of 7%).

However, we expect the consequence of this bias to be negligible. First, in only a fraction of the cases where a child was sampled would the father have otherwise been sampled. Second, the survey asks men that were selected as the primary respondents how many children they had, not how many they lived with. That is, the average number of resident children per selected possible father is only correlated with our metric of interest. Finally, comparisons with recent annual age-specific male fertility rates estimated from live births data in Supplementary Section 2.3 confirm the impact of this bias is small.

1.2.4 Estimating double orphanhood

As a child may lose both their mother and father, estimates of total orphanhood do not equal the sum of orphanhood from the loss of the mother and orphanhood from the loss of the father. Adjusting for this is non-trivial and requires estimates of the joint fertility rates and mortality rates.

Before estimating these quantities, we denote the male-partner-age-distribution of females in state r by $P_{m|f,r}$. That is, $P_{m|f,r}$ denotes the probability that a female in age-group f and state r has a partner in age-group m, such that, $\forall f, r$, we have $\sum_{m \in M} P_{m|f,r} = 1$, where M is the set of male age-groups. $P_{m|f,r}$ is estimated from PNS survey data by constructing a *svydesign* object, subsetting by each combination of f, r, and using the *svymean()* function to estimate the proportion of partners in each age-group m. Code to reproduce this is given in the `sampleOrphanhoodHelpers.R` script in the GitHub repository.

Let $E'_{f,m,r,y}$ represent the number of occurrences where a mother in age-group f and the corresponding father in age-group m both die. We generate samples of this quantity by assuming that partner deaths are independent and:

- Sampling $x \sim \text{Multinomial}(E_{f,\text{female},r,y}, \{P_{m|f,r}\}_m)$
- Sampling $E'_{f,m,r,y} \sim \text{Binomial}(x, E_{m,\text{male},r,y}/N_{m,\text{male},r})$

where $N_{m,\text{male},r}$ is the population size of males in age-group m and state r. The term $E_{m,\text{male},r,y}/N_{m,\text{male},r}$ is the probability that a randomly selected male in age-group m and state r has died. As this probability is generally small, the binomial sampler here is an appropriate approximation.

There is insufficient information to estimate the number of children aged less than 18 that jointly belong to a mother in age-group m and father in age-group f. As women have a more restricted window of fertility, we simply use the female fertility rate. This ignores the fact that a mother in age-group f is less likely to have children aged ≤ 17 if their partner is older, which is also when both parents are more likely to have died, so we likely overestimate double orphanhood. Our estimates are still very small in absolute terms, so this has negligible impact on overall results.

Thus we generate samples of double orphanhood by setting:

$$O'^{(i)}_{f,m,r,y} = \sum_{j=1}^{E'^{(i)}_{f,m,r,y}} C^{(j)}_{a,\text{female},r}$$

This is calculated at the same time as $O^{(i)}_{a,s,r,y}$ (for $s \in \{\text{female}, \text{male}\}$) so relies upon the same samples of mortality as single parent orphanhood. We report single-parent orphanhood with double orphanhood subtracted, so total orphanhood is the sum of single-parent orphanhood and double orphanhood.

1.2.5 Estimating the loss of co-residing grandparents and older kin

The PNS 2019 survey also allows us to consider household-based estimates of orphanhood, meaning we can estimate the number of children that lost a co-residing grandparent or older kin. The statistical approach for estimating this differs to the approach we employ for estimating orphanhood.

We use mortality data to estimate the probability that any single individual in age-group a, sex s, and state r died. We calculate this for the ith sample of mortality $E^{(i)}_{a,s,r}$ as:

$$p^{(i)}_{a,s,r} = \frac{E^{(i)}_{a,s,r}}{N_{a,s,r}}$$

Each individual listed in the PNS survey is then labelled as having died with probability $p^{(i)}_{a,s,r}$. When estimating orphanhood due to all-cause mortality, there is no uncertainty in the mortality rates, so $p_{a,s,r}$ is deterministic. We still randomly re-allocate deaths to individuals at each iteration, allowing for uncertainty in who died.

Grouping by household ID, we then calculate the number of households where an elderly person died. Additional quantities are also calculated, such as whether an elderly person died in households with no adults aged 18-59, or whether multiple elderly people died. This is used to augment the survey data, creating a new variable for all individuals in each household that denotes whether an elderly person died in that household.

Filtering the survey data so that only children aged 0-to-17 are considered, the *svyciprop()* function from the *survey* package [11] is used to estimate the proportion of children in each region that lived in a household where an elderly person died (or in a household matching the other conditions). This function takes a confidence level as an argument, so we use the inversion method to generate samples from the implied distribution for the proportion of children living in impacted households.

Specifically, we sample $u_i \sim U(0, 1)$ and set:

$$\text{level} = \begin{cases} 1 - 2u_i & \text{if } u_i \leq 0.5 \\ 2u_i - 1 & \text{if } u_i > 0.5 \end{cases}$$

Let (l_i, u_i) be the confidence interval on the proportion of children in region r living in a household where an elderly person died at the determined confidence *level*. The ith sample of the proportion of children living in a household where an elderly person died is then multiplied by the population of children n_r to give orphanhood estimates associated with the death of co-resident elderly people:

$$O_r^{(i)} = \begin{cases} n_r l_i & \text{if } u_i \leq 0.5 \\ n_r u_i & \text{if } u_i > 0.5 \end{cases}$$

It is worth noting that our uncertainty intervals are wider than necessary, as using mortality estimates to calculate the probability that an individual died means we are not conditioning on total mortality. Future work could decrease the width of these intervals by ensuring the implied total mortality is equal to the observed total mortality (at each iteration).

1.2.6 Estimating the number of children experiencing either parental orphanhood and the loss of co-residing grandparents and older kin

To produce estimates of the number of children experiencing at least one of the two types of orphanhood considered, we need to quantify the number of children experiencing both types of orphanhood. These numbers are small in absolute terms, so we use a PNS-survey-based approximation in order to save on computation time. Specifically, we assign deaths to individuals in the survey data as per Supplementary Section 1.2.5, and calculate the proportion of children (in each state) that lived in a household where both an adult aged 20-59 and an adult aged 60+ died. This process is repeated 1000 times, generating samples of the proportion of children that experienced both types of orphanhood considered.

In extending this analysis to the population of Brazil we make three main assumptions. First, we assume that the unweighted survey data are representative of Brazil at the state-level. This assumption is unlikely to hold, but given the very small numbers considered and large statistical noise, will have no practical effect on estimated totals. Second, we assume that adults living in households with children are always the parents. Again this is unlikely to hold in practice, but bias introduced by this assumption is negligible compared to other sources of statistical noise. Finally, we assume that deaths of parents and co-residing elderly people are independent.

1.2.7 Estimating monthly orphanhood

Uncertainty associated with monthly excess mortality was unavailable, so we can only present central estimates, and thus the samples-based workflow is ignored in favour of simpler calculations. Letting $E_{a,s,r,m,y}$ be the mortality (excess or all-cause) in adults of age-group a, sex s, state r, month m, and year y, and $C_{a,s,r}$ be the corresponding mean number of children born to adults in this group, then central estimates of orphanhood in month m of year y are given by:

$$O_{a,s,r,m,y} = E_{a,s,r,m,y} C_{a,s,r}$$

$C_{a,s,r}$ is estimated by taking the mean of the individual samples generated in Supplementary

Sections 1.2.2 and 1.2.3. This assumes that the average number of children born to parents in each group is constant over the two years considered.

1.2.8 Structure of code

We provide a flowchart demonstrating the structure of the code in Supplementary Figure 5.

2 Supplementary results

2.1 Children experiencing both parental orphanhood and the loss of co-residing grandparents and older kin

Estimates of the number of children experiencing the loss of both a parent and an elderly caregiver are provided, by federative unit, in Supplementary Table 1.

2.2 Regional orphanhood incidence by child age

Estimates of orphanhood per 1000 children by the age-of-child and federative unit are provided in Supplementary Figure 6.

2.3 Annual age-specific male fertility rates

From 2010 onwards, the age of the father was included on some live births records. Only 1.4% of records included these data in 2010, however response rates ranged between 31.9% and 52.5% between 2011 and 2020. While this does not enable us to estimate orphanhood due to the loss of a father (as this would require age-of-father data back to 2003), we can use it to verify our male fertility estimates derived from PNS data.

The birth-year of the oldest child is included in the survey data. The birth-year of the youngest child (where 2 or more children are present) is estimated by $2019 - a$, where a is the age of the youngest child, while middle children are assigned a uniformly distributed age between the oldest and youngest child. For computational reasons (as we only report results from this analysis here, and not in the main text) we only do this once, so uncertainty about age-specific male fertility rates is slightly understated here. As data were collected throughout 2019, estimates for this year are smaller than if they were based on complete data - while this impacts our estimates in this section, they also do not impact other estimates based on aggregate data. Finally, we do not account for child-mortality, introducing a slight downward bias in PNS estimates relative to live-births estimates.

Supplementary Figure 7 shows the PNS-derived estimates of age-specific male fertility rates and corresponding confidence intervals for 10-year age-groups. The figure also shows estimates derived from live births data, assuming that data with age-of-father included are representative of data with age-of-father missing.

The two methods produce largely consistent output, with uncertainty associated with PNS-derived estimates generally including estimates derived from live births data.

2.4 Age-distribution of children listed in the Campinas administrative data

Of the 481 children listed in the administrative data from Campinas, 375 also had their age listed. We calculate the proportion of these children that were of each age and compare this to the estimated age-distribution of orphanhood in São Paulo state in Supplementary Figure 8. We also present approximate 95% confidence intervals for the Campinas estimates using the Sison and Glaz method, although as we expect the administrative data to contain a sizeable

proportion of orphanhood over this period, these confidence intervals are likely wider than they should be.

Both sources of data demonstrate that older children are more likely to experience orphanhood in São Paulo. Our central estimates exhibit a slightly steeper age-gradient than those derived from the Campinas data, which may be a result of differences between Campinas and the rest of São Paulo, biases in our method, or biases in the collection of the administrative data.

2.5 Orphanhood estimates by region

In the main manuscript, we present estimates of orphanhood by federative unit. In addition to this spatial stratification, Brazil is often stratified into five regions: North, Northeast, Southeast, South, and Central-West. To aid interpretation of our results, we also present summary statistics (mean and 95% uncertainty intervals) of orphanhood estimates at these stratifications. Supplementary Tables 2 and 3 present these estimates for all-cause and excess mortality-derived estimates respectively.

3 Supplementary figures

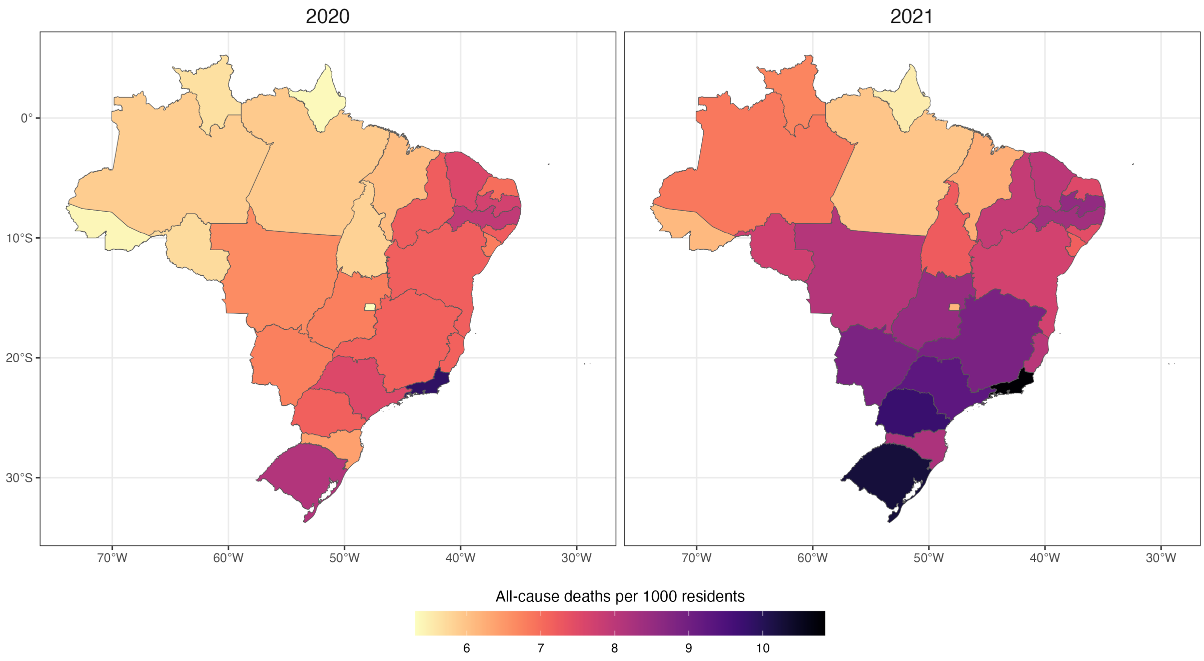

Supplementary Figure 1: All-cause mortality by federative unit for 2020 and 2021.

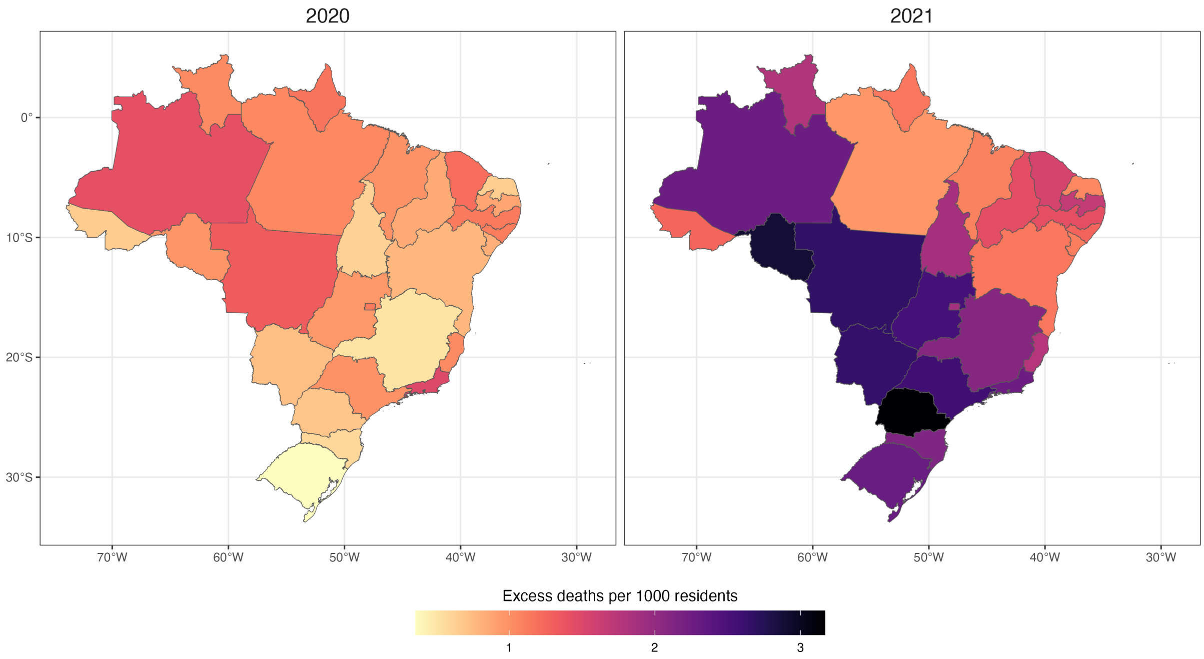

Supplementary Figure 2: Excess mortality estimates by federative unit for 2020 and 2021.

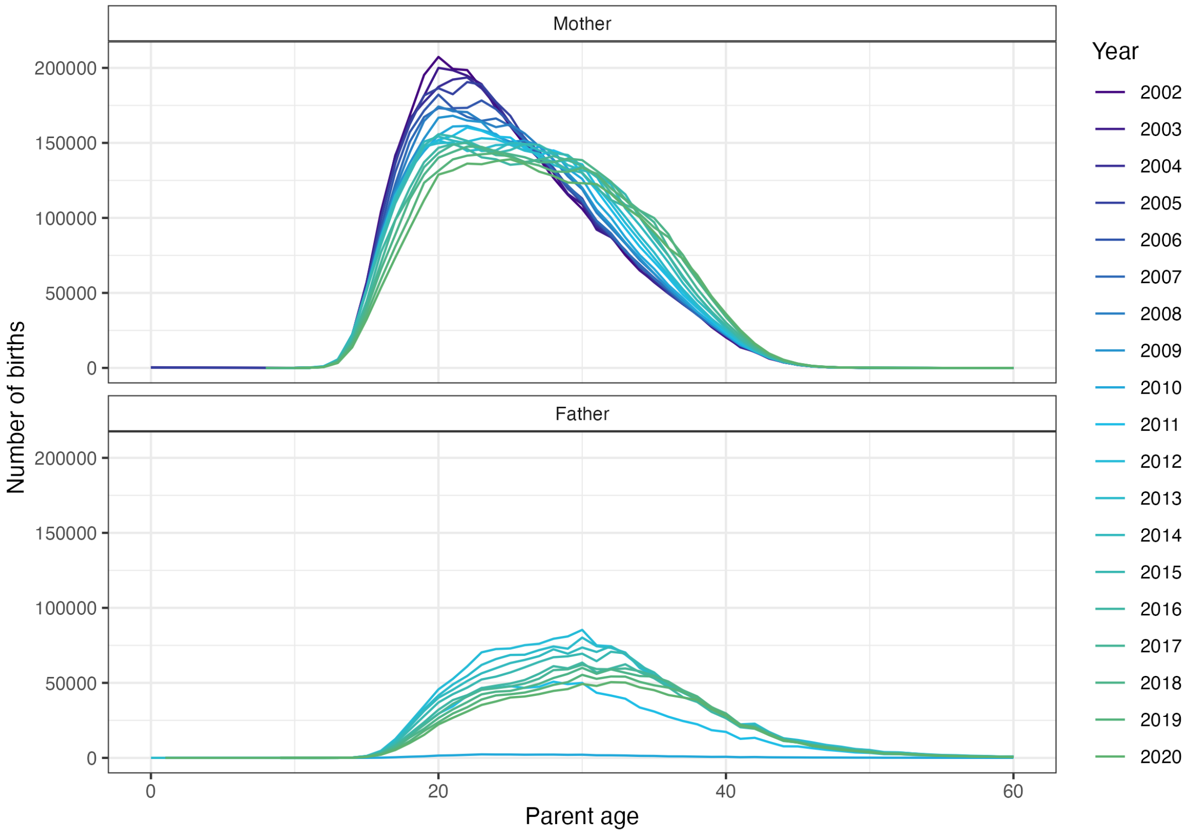

Supplementary Figure 3: Annual reported live births by parent sex and age calculated from live births line data. The age-of-father was recorded from 2010 onwards, although response rates range between 31.9% and 52.5% (between 2011 and 2020) and 1.4% in 2010. The line near the x-axis in the father sub-panel are the number of births recorded to fathers in 2010.

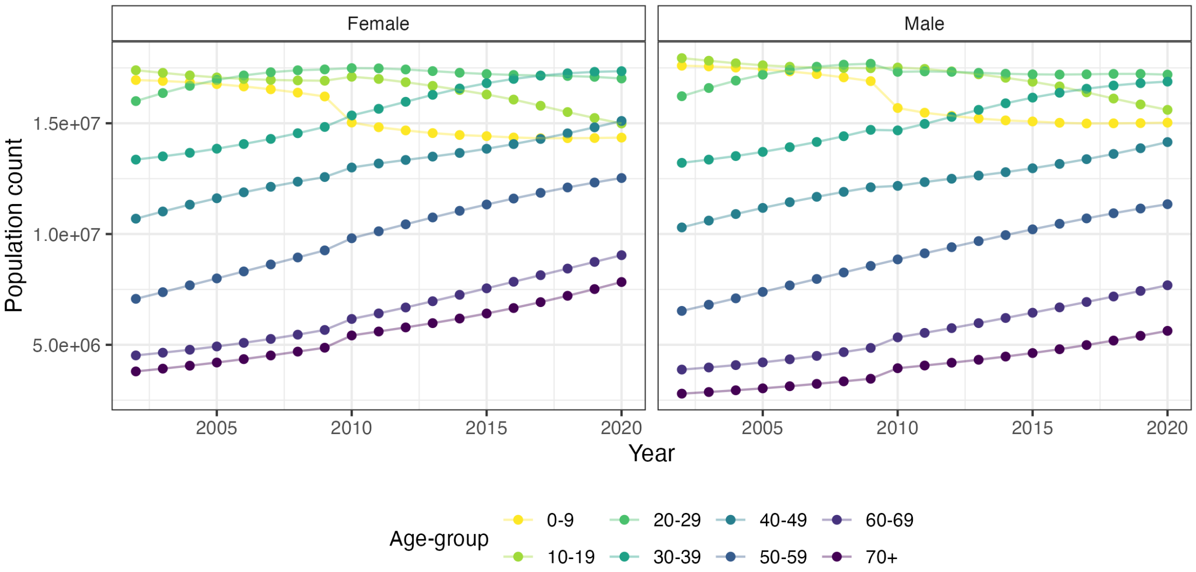

Supplementary Figure 4: Population estimates for Brazil by sex and age-group from 2003 to 2020. Note the small discontinuity from 2009 to 2010 as the source of population data changes.

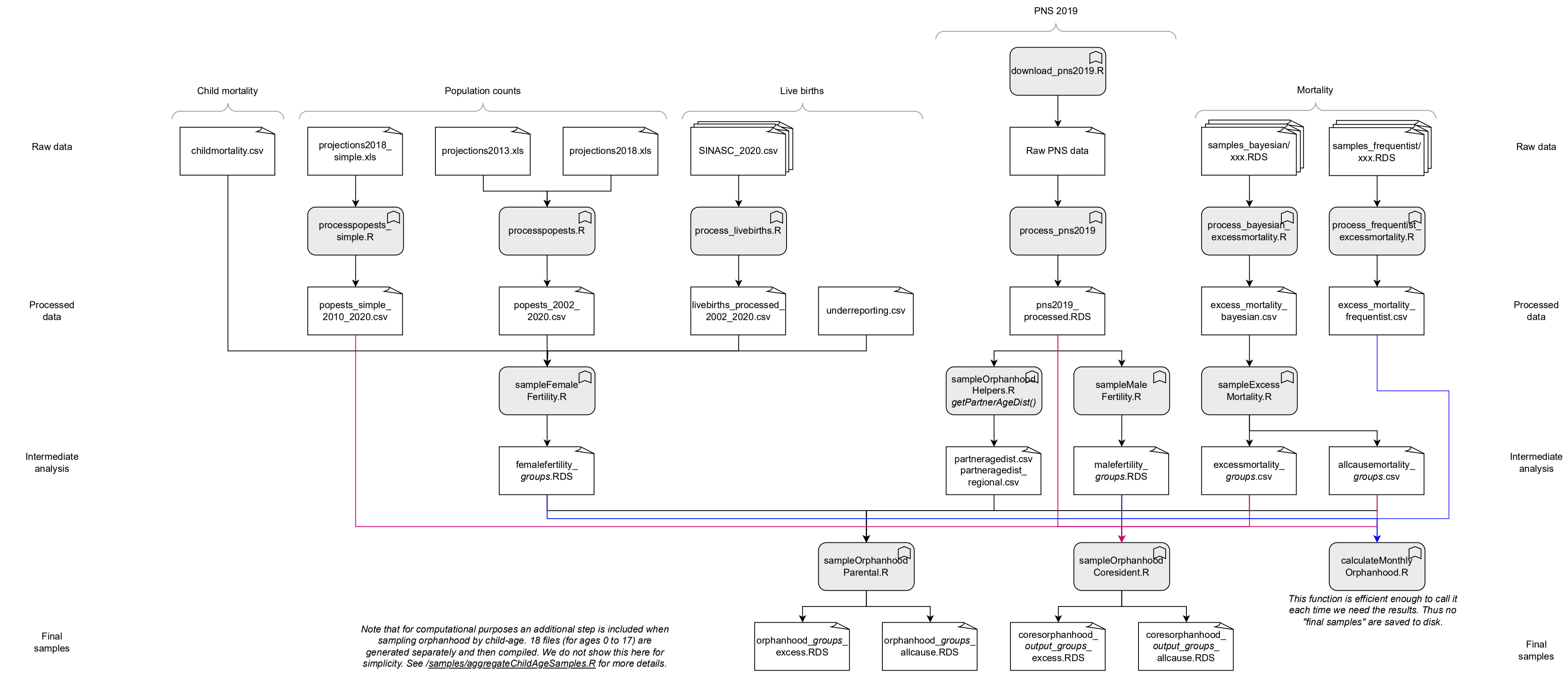

Supplementary Figure 5: A flowchart demonstrating the structure of the code used to produce the final set of samples of orphanhood. Boxes with rounded edges represent R scripts, while boxes with square edges represent files.

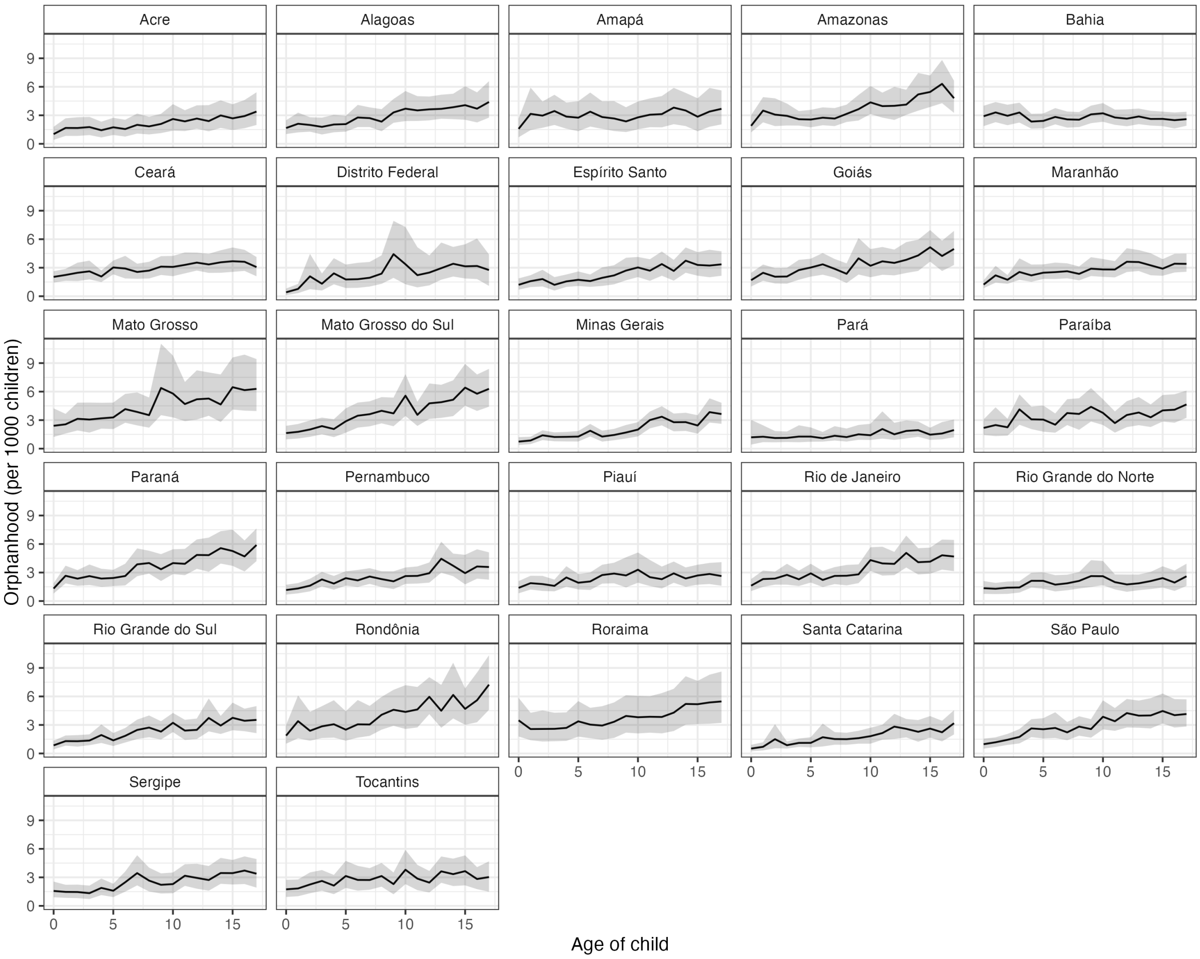

Supplementary Figure 6: Orphanhood per 1000 children by age-of-child and federative unit.

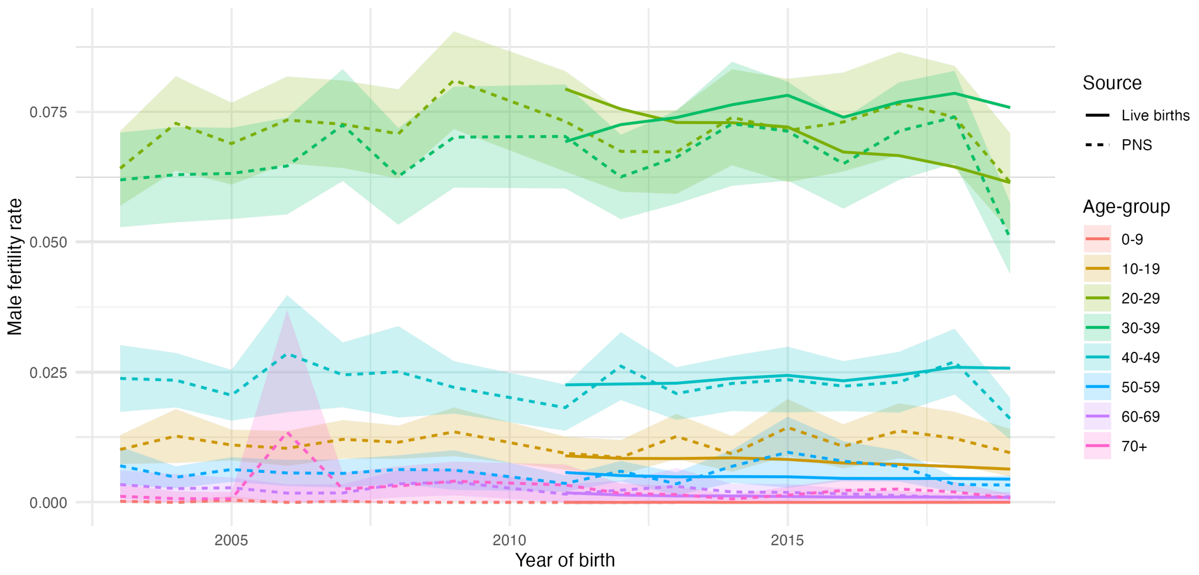

Supplementary Figure 7: A comparison of PNS-derived estimates of annual age-specific male fertility rates (dashed lines and shaded regions) with those derived from live births data (solid lines). This shows that our PNS-derived estimates are similar to the gold-standard live births data, when the live births data were available for comparison. PNS is the National Survey of Health (Pesquisa Nacional de Saúde) in Brazil.

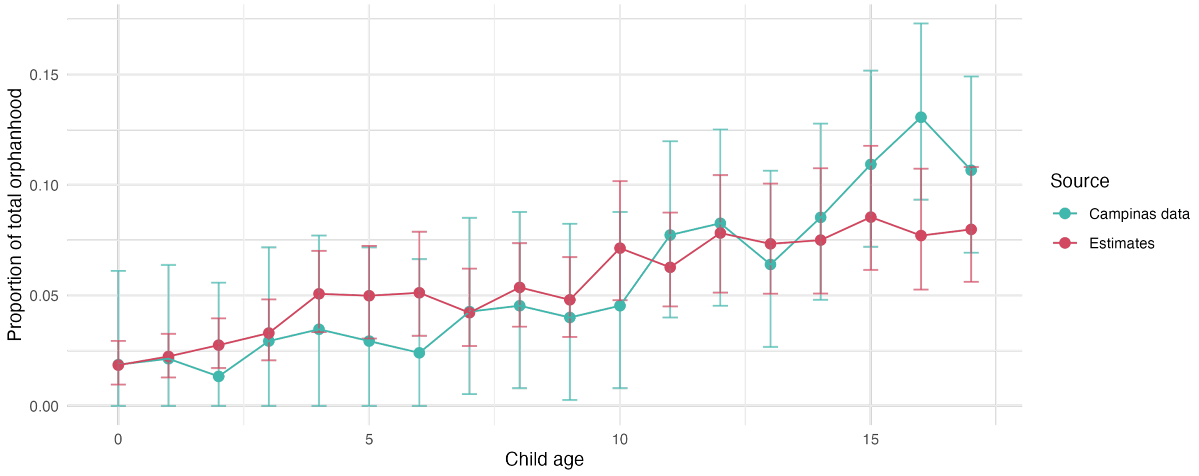

Supplementary Figure 8: The estimated age distribution of orphanhood in São Paulo using our methods (red) and the estimated age distribution of orphanhood in Campinas, a municipality of São Paulo, using administrative data (blue).

4 Supplementary tables

Supplementary Table 1: Estimates of the number of children experiencing the loss of both a parent and an elderly caregiver by federative unit. Uncertainty intervals on COVID-19-associated estimates often do not include the mean estimate due to many samples featuring no instances of a child that loses both a parent and elderly caregiver (see Supplementary Section 1.2.6 for further details).

	COVID-19 associated		All-cause	
Region	Total	Per 100000	Total	Per 100000
Acre	1.27 (0, 0)	0.4 (0, 0)	48.9 (0, 381)	15.7 (0, 122)
Alagoas	7.09 (0, 0)	0.7 (0, 0)	201 (0, 1420)	20.6 (0, 145.8)
Amapá	3.01 (0, 0)	1 (0, 0)	77.7 (0, 574)	26.7 (0, 196.9)
Amazonas	31.3 (0, 330)	2.2 (0, 22.9)	370 (0, 2310)	25.7 (0, 160.4)
Bahia	1.51 (0, 0)	0 (0, 0)	568 (0, 4540)	14.6 (0, 116.9)
Ceará	26.7 (0, 666)	1.1 (0, 27.8)	492 (0, 2670)	20.5 (0, 111.1)
Distrito Federal	3.16 (0, 0)	0.4 (0, 0)	77.1 (0, 901)	10.4 (0, 121.5)
Espírito Santo	3.43 (0, 0)	0.3 (0, 0)	124 (0, 858)	12.2 (0, 84.4)
Goiás	17.4 (0, 0)	0.9 (0, 0)	264 (0, 1940)	14.3 (0, 105.4)
Maranhão	22.2 (0, 412)	1 (0, 18.4)	470 (0, 2470)	21 (0, 110.2)
Mato Grosso	19.6 (0, 0)	2 (0, 0)	166 (0, 1590)	16.9 (0, 161.8)
Mato Grosso do Sul	9.97 (0, 0)	1.3 (0, 0)	121 (0, 1070)	15.9 (0, 139.9)
Minas Gerais	18.3 (0, 0)	0.4 (0, 0)	588 (0, 4580)	12.1 (0, 94.2)
Pará	10.5 (0, 0)	0.4 (0, 0)	627 (0, 3490)	23.1 (0, 128.3)
Paraíba	3.94 (0, 0)	0.4 (0, 0)	245 (0, 1320)	23.3 (0, 125.5)
Paraná	4.28 (0, 0)	0.2 (0, 0)	286 (0, 2140)	10.3 (0, 77)
Pernambuco	12 (0, 0)	0.5 (0, 0)	481 (0, 2580)	18.6 (0, 99.8)
Piauí	4.25 (0, 0)	0.5 (0, 0)	198 (0, 1280)	22 (0, 142.2)
Rio de Janeiro	41.1 (0, 0)	1 (0, 0)	892 (0, 5670)	22.6 (0, 143.7)
Rio Grande do Norte	4.35 (0, 0)	0.5 (0, 0)	213 (0, 1580)	23.7 (0, 175.4)
Rio Grande do Sul	7.47 (0, 0)	0.3 (0, 0)	328 (0, 2490)	13 (0, 98.8)
Rondônia	9.81 (0, 0)	2 (0, 0)	78.8 (0, 755)	15.7 (0, 150.9)
Roraima	3.05 (0, 0)	1.5 (0, 0)	50.3 (0, 305)	25.1 (0, 152.4)
Santa Catarina	6.62 (0, 0)	0.4 (0, 0)	143 (0, 1660)	8.6 (0, 98.9)
São Paulo	80.3 (0, 0)	0.7 (0, 0)	1510 (0, 8600)	13.9 (0, 79.1)
Sergipe	0.628 (0, 0)	0.1 (0, 0)	100 (0, 941)	15.9 (0, 149.4)
Tocantins	2.49 (0, 0)	0.5 (0, 0)	82.3 (0, 831)	17.7 (0, 178.7)

Supplementary Table 2: Estimates of orphanhood due to any cause of parental death and estimates of the number of children that lost a co-residing grandparent or other older kin. Estimates are reported as central estimates with 95% uncertainty intervals in parentheses. This table is functionally equivalent to Table 1 in the main manuscript, except with results aggregated at a different geographical stratification.

**Region**	**Total**	**Per 1000 children**
	All-cause orphanhood	
North	74500 (71300, 78700)	12.6 (12, 13.3)
Northeast	208000 (200000, 217000)	13.4 (12.8, 13.9)
Southeast	254000 (239000, 268000)	12.3 (11.5, 13)
South	81400 (77000, 86500)	11.7 (11, 12.4)
Central-West	54400 (51300, 58600)	12.6 (11.9, 13.5)
Total	673000 (652000, 690000)	12.6 (12.2, 12.9)
	All-cause loss of grandparents and older kin	
North	84100 (58500, 114000)	14.2 (9.9, 19.3)
Northeast	216000 (159000, 289000)	13.9 (10.2, 18.6)
Southeast	228000 (153000, 322000)	11 (7.4, 15.6)
South	64700 (36000, 103000)	9.3 (5.2, 14.8)
Central-West	42300 (24800, 66700)	9.8 (5.7, 15.4)
Total	635000 (534000, 758000)	11.9 (10, 14.2)
	All-cause loss of parents and/or older kin	
North	157000 (132000, 188000)	26.5 (22.2, 31.7)
Northeast	422000 (367000, 495000)	27.1 (23.5, 31.8)
Southeast	478000 (403000, 575000)	23.1 (19.5, 27.8)
South	145000 (116000, 184000)	20.8 (16.7, 26.4)
Central-West	96000 (78100, 121000)	22.2 (18.1, 27.9)
Total	1300000 (1190000, 1430000)	24.3 (22.3, 26.7)

Supplementary Table 3: Estimates of orphanhood due to coronavirus disease 2019 (COVID-19)-associated deaths and estimates of the number of children that lost a co-residing grandparent or other older kin. Estimates are reported as central estimates with 95% uncertainty intervals in parentheses. This table is functionally equivalent to Table 2 in the main manuscript, except with results aggregated at a different geographical stratification.

**Region**	**Total**	**Per 1000 children**
COVID-19-associated orphanhood		
North	14900 (13800, 16100)	2.5 (2.3, 2.7)
Northeast	42800 (40800, 44800)	2.8 (2.6, 2.9)
Southeast	57500 (53000, 61300)	2.8 (2.6, 3)
South	18800 (17500, 20100)	2.7 (2.5, 2.9)
Central-West	15100 (14000, 16400)	3.5 (3.2, 3.8)
Total	149000 (144000, 154000)	2.8 (2.7, 2.9)
COVID-19-associated loss of grandparents and older kin		
North	23700 (12100, 41300)	4 (2, 7)
Northeast	37500 (18300, 72900)	2.4 (1.2, 4.7)
Southeast	47900 (15700, 101000)	2.3 (0.8, 4.9)
South	14300 (2760, 34500)	2 (0.4, 5)
Central-West	12000 (2980, 29700)	2.8 (0.7, 6.9)
Total	135000 (85900, 199000)	2.5 (1.6, 3.7)
COVID-19-associated loss of parents and/or older kin		
North	38500 (26800, 56400)	6.5 (4.5, 9.5)
Northeast	80300 (61200, 114000)	5.2 (3.9, 7.3)
Southeast	105000 (73500, 159000)	5.1 (3.6, 7.7)
South	33000 (21300, 54200)	4.7 (3.1, 7.8)
Central-West	27000 (17700, 44900)	6.2 (4.1, 10.4)
Total	284000 (235000, 348000)	5.3 (4.4, 6.5)

Supplementary Table 4: Estimates of orphanhood due to any cause of parental death by sex of parent and federative unit. Double orphanhood refers to the loss of both parents.

Region	Maternal	Paternal	Double	Total	Maternal (per 1000)	Paternal (per 1000)	Double (per 1000)	Total (per 1000)
Acre	1030 (917, 1160)	2840 (2470, 3300)	17.7 (7.98, 30)	3890 (3470, 4370)	3.3 (2.9, 3.7)	9.1 (7.9, 10.5)	0.1 (0, 0.1)	12.5 (11.1, 14)
Alagoas	2920 (2580, 3310)	10200 (9100, 11700)	56.6 (35, 79)	13200 (12000, 14800)	3 (2.7, 3.4)	10.5 (9.4, 12)	0.1 (0, 0.1)	13.5 (12.4, 15.2)
Amapá	974 (861, 1090)	2920 (2440, 3480)	15.1 (6, 27)	3910 (3410, 4470)	3.3 (3, 3.7)	10 (8.4, 11.9)	0.1 (0, 0.1)	13.4 (11.7, 15.3)
Amazonas	4530 (4080, 5010)	15400 (13400, 17200)	80.5 (54, 111)	20000 (18000, 21800)	3.2 (2.8, 3.5)	10.7 (9.3, 12)	0.1 (0, 0.1)	13.9 (12.5, 15.1)
Bahia	11300 (9870, 13100)	39300 (34000, 44700)	168 (126, 219)	50900 (45500, 56100)	2.9 (2.5, 3.4)	10.1 (8.8, 11.5)	0 (0, 0.1)	13.1 (11.7, 14.4)
Ceará	7150 (6120, 8170)	24500 (21900, 27700)	115 (80, 159)	31800 (29000, 35000)	3 (2.6, 3.4)	10.2 (9.1, 11.5)	0 (0, 0.1)	13.3 (12.1, 14.6)
Distrito Federal	2150 (1870, 2400)	5810 (4770, 7080)	26.6 (14, 42)	7980 (6940, 9290)	2.9 (2.5, 3.2)	7.8 (6.4, 9.5)	0 (0, 0.1)	10.8 (9.4, 12.5)
Espírito Santo	3130 (2730, 3550)	9020 (7800, 10300)	42.5 (25, 62)	12200 (11000, 13500)	3.1 (2.7, 3.5)	8.9 (7.7, 10.2)	0 (0, 0.1)	12 (10.8, 13.3)
Goiás	5900 (5240, 6710)	16900 (15000, 19200)	89.9 (62, 120)	22800 (20800, 25300)	3.2 (2.9, 3.6)	9.2 (8.1, 10.4)	0 (0, 0.1)	12.4 (11.3, 13.8)
Maranhão	5480 (4790, 6080)	23000 (20100, 26200)	83.3 (58, 113)	28500 (25500, 31900)	2.4 (2.1, 2.7)	10.3 (9, 11.7)	0 (0, 0.1)	12.7 (11.4, 14.2)
Mato Grosso	3460 (2980, 3880)	9690 (7970, 13000)	55.9 (34, 80)	13200 (11300, 16500)	3.5 (3, 3.9)	9.9 (8.1, 13.3)	0.1 (0, 0.1)	13.4 (11.5, 16.8)
Mato Grosso do Sul	2810 (2460, 3240)	7470 (6700, 8310)	42.4 (26, 64)	10300 (9440, 11300)	3.7 (3.2, 4.2)	9.8 (8.8, 10.9)	0.1 (0, 0.1)	13.5 (12.4, 14.7)
Minas Gerais	15100 (13200, 17600)	37200 (34000, 40200)	224 (169, 307)	52500 (48600, 56300)	3.1 (2.7, 3.6)	7.6 (7, 8.3)	0 (0, 0.1)	10.8 (10, 11.6)
Paraná	9590 (8440, 10700)	25000 (22300, 27800)	151 (113, 191)	34700 (31600, 37700)	3.4 (3, 3.8)	9 (8, 10)	0.1 (0, 0.1)	12.5 (11.4, 13.6)
Paraíba	3280 (2840, 3780)	11900 (10500, 13700)	56 (35, 82)	15200 (13800, 17100)	3.1 (2.7, 3.6)	11.3 (10, 13)	0.1 (0, 0.1)	14.4 (13.1, 16.2)
Pará	6940 (6250, 7640)	23500 (20800, 27100)	91.5 (63, 123)	30600 (27600, 34200)	2.6 (2.3, 2.8)	8.7 (7.7, 10)	0 (0, 0)	11.2 (10.1, 12.6)
Pernambuco	7920 (7070, 8800)	27300 (23900, 30900)	137 (101, 176)	35300 (31800, 38900)	3.1 (2.7, 3.4)	10.6 (9.3, 12)	0.1 (0, 0.1)	13.7 (12.3, 15.1)
Piauí	2440 (2070, 2780)	8660 (7430, 9890)	35 (19, 52)	11100 (9900, 12400)	2.7 (2.3, 3.1)	9.7 (8.3, 11)	0 (0, 0.1)	12.4 (11, 13.8)
Rio Grande do Norte	2490 (2180, 2780)	10000 (8580, 12600)	34.4 (19, 52)	12600 (11100, 15100)	2.8 (2.4, 3.1)	11.1 (9.5, 14)	0 (0, 0.1)	13.9 (12.3, 16.8)
Rio Grande do Sul	8870 (7460, 10300)	21700 (19100, 24600)	134 (95, 180)	30700 (27900, 33900)	3.5 (3, 4.1)	8.6 (7.6, 9.8)	0.1 (0, 0.1)	12.2 (11.1, 13.5)
Rio de Janeiro	17100 (14500, 19700)	38400 (34700, 42600)	307 (235, 400)	55800 (51300, 60400)	4.3 (3.7, 5)	9.7 (8.8, 10.8)	0.1 (0.1, 0.1)	14.1 (13, 15.3)
Rondônia	1450 (1260, 1660)	5730 (4760, 7030)	22.4 (11, 37)	7200 (6200, 8500)	2.9 (2.5, 3.3)	11.5 (9.5, 14.1)	0 (0, 0.1)	14.4 (12.4, 17)
Roraima	870 (762, 970)	2620 (2260, 3250)	13.7 (4, 26)	3500 (3130, 4130)	4.3 (3.8, 4.8)	13.1 (11.3, 16.2)	0.1 (0, 0.1)	17.5 (15.6, 20.6)
Santa Catarina	5010 (4420, 5760)	10900 (9700, 12100)	60.6 (39, 86)	16000 (14600, 17400)	3 (2.6, 3.4)	6.5 (5.8, 7.2)	0 (0, 0.1)	9.5 (8.7, 10.4)
Sergipe	1860 (1650, 2070)	7990 (7050, 9290)	34.4 (21, 52)	9890 (8900, 11200)	3 (2.6, 3.3)	12.7 (11.2, 14.7)	0.1 (0, 0.1)	15.7 (14.1, 17.8)
São Paulo	38400 (33500, 43400)	94100 (81600, 107000)	612 (483, 747)	133000 (119000, 146000)	3.5 (3.1, 4)	8.7 (7.5, 9.9)	0.1 (0, 0.1)	12.2 (10.9, 13.5)
Tocantins	1340 (1180, 1540)	4110 (3490, 4700)	18.6 (8, 31)	5470 (4850, 6080)	2.9 (2.5, 3.3)	8.8 (7.5, 10.1)	0 (0, 0.1)	11.8 (10.4, 13.1)
Total	174000 (166000, 180000)	496000 (475000, 512000)	2730 (2510, 2930)	673000 (652000, 690000)	3.2 (3.1, 3.4)	9.3 (8.9, 9.6)	0.1 (0, 0.1)	12.6 (12.2, 12.9)

Supplementary Table 5: Estimates of orphanhood due to COVID-19-associated parental death by sex of parent and federative unit. Double orphanhood refers to the loss of both parents.

Region	Maternal	Paternal	Double	Total	Maternal (per 1000)	Paternal (per 1000)	Double (per 1000)	Total (per 1000)
Acre	303 (221, 394)	385 (246, 543)	0.737 (0, 4)	689 (534, 859)	1 (0.7, 1.3)	1.2 (0.8, 1.7)	0 (0, 0)	2.2 (1.7, 2.7)
Alagoas	548 (421, 689)	2520 (2170, 2940)	2.76 (0, 8)	3070 (2690, 3520)	0.6 (0.4, 0.7)	2.6 (2.2, 3)	0 (0, 0)	3.2 (2.8, 3.6)
Amapá	287 (216, 368)	603 (451, 786)	0.871 (0, 4)	892 (719, 1080)	1 (0.7, 1.3)	2.1 (1.5, 2.7)	0 (0, 0)	3.1 (2.5, 3.7)
Amazonas	1370 (1180, 1590)	3840 (3170, 4500)	7.18 (1, 16)	5220 (4530, 5900)	1 (0.8, 1.1)	2.7 (2.2, 3.1)	0 (0, 0)	3.6 (3.1, 4.1)
Bahia	2690 (2270, 3160)	8150 (7150, 9190)	6.16 (1, 14)	10800 (9830, 12000)	0.7 (0.6, 0.8)	2.1 (1.8, 2.4)	0 (0, 0)	2.8 (2.5, 3.1)
Ceará	1220 (1010, 1470)	5740 (5130, 6430)	4.09 (0, 10)	6970 (6340, 7700)	0.5 (0.4, 0.6)	2.4 (2.1, 2.7)	0 (0, 0)	2.9 (2.6, 3.2)
Distrito Federal	337 (250, 436)	1410 (1090, 1830)	1.29 (0, 5)	1750 (1430, 2180)	0.5 (0.3, 0.6)	1.9 (1.5, 2.5)	0 (0, 0)	2.4 (1.9, 2.9)
Espírito Santo	835 (690, 1010)	1550 (1280, 1870)	2.15 (0, 7)	2390 (2080, 2740)	0.8 (0.7, 1)	1.5 (1.3, 1.8)	0 (0, 0)	2.4 (2, 2.7)
Goiás	1880 (1600, 2210)	4130 (3560, 4860)	7.2 (1, 16)	6010 (5370, 6830)	1 (0.9, 1.2)	2.2 (1.9, 2.6)	0 (0, 0)	3.3 (2.9, 3.7)
Maranhão	1110 (924, 1330)	4850 (4150, 5670)	3.42 (0, 10)	5960 (5210, 6820)	0.5 (0.4, 0.6)	2.2 (1.9, 2.5)	0 (0, 0)	2.7 (2.3, 3)
Mato Grosso	1340 (1140, 1540)	3010 (2490, 3860)	6.6 (1, 15)	4350 (3780, 5230)	1.4 (1.2, 1.6)	3.1 (2.5, 3.9)	0 (0, 0)	4.4 (3.9, 5.3)
Mato Grosso do Sul	1020 (851, 1200)	1910 (1630, 2210)	4.1 (0, 10)	2930 (2600, 3290)	1.3 (1.1, 1.6)	2.5 (2.1, 2.9)	0 (0, 0)	3.8 (3.4, 4.3)
Minas Gerais	3420 (2910, 4030)	6730 (5900, 7450)	10.6 (3, 20)	10200 (9210, 11100)	0.7 (0.6, 0.8)	1.4 (1.2, 1.5)	0 (0, 0)	2.1 (1.9, 2.3)
Paraná	3100 (2690, 3540)	7050 (6270, 7880)	14.7 (5, 26)	10200 (9280, 11100)	1.1 (1, 1.3)	2.5 (2.3, 2.8)	0 (0, 0)	3.7 (3.3, 4)
Paraíba	692 (551, 845)	2800 (2420, 3230)	2.68 (0, 8)	3500 (3070, 3970)	0.7 (0.5, 0.8)	2.7 (2.3, 3.1)	0 (0, 0)	3.3 (2.9, 3.8)
Pará	1320 (1110, 1550)	2610 (1920, 3490)	2.16 (0, 6.02)	3930 (3210, 4820)	0.5 (0.4, 0.6)	1 (0.7, 1.3)	0 (0, 0)	1.4 (1.2, 1.8)
Pernambuco	1610 (1350, 1880)	5260 (4520, 6000)	5.69 (1, 13)	6880 (6100, 7710)	0.6 (0.5, 0.7)	2 (1.8, 2.3)	0 (0, 0)	2.7 (2.4, 3)
Piauí	410 (292, 532)	1750 (1460, 2060)	0.995 (0, 5)	2170 (1830, 2510)	0.5 (0.3, 0.6)	2 (1.6, 2.3)	0 (0, 0)	2.4 (2, 2.8)
Rio Grande do Norte	417 (315, 534)	1380 (1090, 1730)	0.772 (0, 4)	1800 (1500, 2160)	0.5 (0.3, 0.6)	1.5 (1.2, 1.9)	0 (0, 0)	2 (1.7, 2.4)
Rio Grande do Sul	2250 (1840, 2630)	3640 (3020, 4300)	5.98 (1, 13)	5900 (5180, 6670)	0.9 (0.7, 1)	1.4 (1.2, 1.7)	0 (0, 0)	2.3 (2.1, 2.6)
Rio de Janeiro	5570 (4790, 6500)	7270 (6420, 8120)	22 (11, 36)	12900 (11600, 14100)	1.4 (1.2, 1.6)	1.8 (1.6, 2.1)	0 (0, 0)	3.3 (2.9, 3.6)
Rondônia	426 (334, 511)	1730 (1380, 2200)	2.03 (0, 7)	2160 (1800, 2630)	0.9 (0.7, 1)	3.5 (2.8, 4.4)	0 (0, 0)	4.3 (3.6, 5.3)
Roraima	261 (189, 333)	484 (333, 655)	0.801 (0, 4)	745 (566, 924)	1.3 (0.9, 1.7)	2.4 (1.7, 3.3)	0 (0, 0)	3.7 (2.8, 4.6)
Santa Catarina	1030 (857, 1250)	1680 (1400, 2010)	2.64 (0, 7)	2720 (2400, 3110)	0.6 (0.5, 0.7)	1 (0.8, 1.2)	0 (0, 0)	1.6 (1.4, 1.9)
Sergipe	247 (166, 338)	1380 (1120, 1680)	0.916 (0, 4)	1630 (1350, 1940)	0.4 (0.3, 0.5)	2.2 (1.8, 2.7)	0 (0, 0)	2.6 (2.1, 3.1)
São Paulo	9730 (8400, 11100)	22300 (18400, 25200)	41.8 (25, 63)	32100 (27700, 35600)	0.9 (0.8, 1)	2.1 (1.7, 2.3)	0 (0, 0)	3 (2.5, 3.3)
Tocantins	373 (286, 464)	897 (714, 1100)	0.967 (0, 5)	1270 (1070, 1480)	0.8 (0.6, 1)	1.9 (1.5, 2.4)	0 (0, 0)	2.7 (2.3, 3.2)
Total	43800 (41600, 45800)	105000 (1e+05, 109000)	161 (128, 195)	149000 (144000, 154000)	0.8 (0.8, 0.9)	2 (1.9, 2)	0 (0, 0)	2.8 (2.7, 2.9)

Supplementary Table 6: Estimates of the number of children that lost a co-residing grandparent or elderly kin due to any cause. Single refers to the loss of a single co-residing grandparent or elderly kin, multiple refers to the loss of more than 1, any refers to the loss of one or multiple, and only refers to the loss of a co-residing grandparent or elderly kin where there were no adults aged 18-64 in the household.

Region	Single	Multiple	Any	Only	Single (per 1000)	Multiple (per 1000)	Any (per 1000)	Only (per 1000)
Acre	3650 (1350, 6920)	69 (0, 700)	3700 (1460, 7030)	657 (31, 2070)	11.7 (4.3, 22.1)	0.2 (0, 2.2)	11.8 (4.7, 22.5)	2.1 (0.1, 6.6)
Alagoas	14800 (4620, 27200)	362 (0, 2510)	14900 (0, 28600)	2530 (263, 7530)	15.2 (4.7, 28)	0.4 (0, 2.6)	15.4 (0, 29.4)	2.6 (0.3, 7.7)
Amapá	3490 (0, 8220)	133 (0, 1140)	3530 (0, 7580)	511 (0, 2380)	12 (0, 28.2)	0.5 (0, 3.9)	12.1 (0, 26)	1.8 (0, 8.2)
Amazonas	24300 (12600, 38900)	771 (0, 4950)	24500 (13500, 39500)	2400 (225, 6880)	16.9 (8.8, 27)	0.5 (0, 3.4)	17.1 (9.4, 27.5)	1.7 (0.2, 4.8)
Bahia	48600 (15100, 111000)	1280 (0, 12300)	48200 (15100, 102000)	10200 (0, 46800)	12.5 (3.9, 28.6)	0.3 (0, 3.2)	12.4 (3.9, 26.4)	2.6 (0, 12.1)
Ceará	34500 (17600, 57600)	794 (0, 4910)	34700 (18400, 57800)	4580 (545, 13300)	14.4 (7.3, 24)	0.3 (0, 2)	14.4 (7.7, 24.1)	1.9 (0.2, 5.6)
Distrito Federal	6060 (0, 13800)	204 (0, 1470)	6090 (0, 13500)	257 (0, 2340)	8.2 (0, 18.6)	0.3 (0, 2)	8.2 (0, 18.2)	0.3 (0, 3.2)
Espírito Santo	8530 (3050, 17400)	183 (0, 1640)	8540 (3160, 16800)	1020 (0, 3730)	8.4 (3, 17.1)	0.2 (0, 1.6)	8.4 (3.1, 16.5)	1 (0, 3.7)
Goiás	17100 (5780, 37700)	558 (0, 4980)	17500 (6370, 36800)	3440 (0, 13200)	9.3 (3.1, 20.5)	0.3 (0, 2.7)	9.5 (3.5, 20)	1.9 (0, 7.2)
Maranhão	36600 (20900, 55800)	828 (0, 4980)	37100 (21000, 56400)	5500 (1560, 12800)	16.3 (9.3, 24.9)	0.4 (0, 2.2)	16.5 (9.4, 25.2)	2.5 (0.7, 5.7)
Mato Grosso	9530 (1920, 22200)	345 (0, 2580)	9760 (2340, 22400)	1470 (0, 8350)	9.7 (2, 22.6)	0.4 (0, 2.6)	9.9 (2.4, 22.8)	1.5 (0, 8.5)
Mato Grosso do Sul	8860 (2940, 17900)	232 (0, 2250)	8920 (2300, 17600)	1550 (0, 5190)	11.6 (3.8, 23.4)	0.3 (0, 2.9)	11.7 (3, 23)	2 (0, 6.8)
Minas Gerais	51000 (20400, 94400)	927 (0, 8730)	52200 (21400, 101000)	6350 (0, 23400)	10.5 (4.2, 19.4)	0.2 (0, 1.8)	10.7 (4.4, 20.8)	1.3 (0, 4.8)
Paraná	25800 (9160, 50400)	473 (0, 3920)	25700 (8390, 50000)	3680 (0, 12500)	9.3 (3.3, 18.1)	0.2 (0, 1.4)	9.2 (3, 18)	1.3 (0, 4.5)
Paraíba	15500 (0, 28100)	514 (0, 3730)	15800 (0, 29400)	2010 (116, 6080)	14.7 (0, 26.6)	0.5 (0, 3.5)	15 (0, 27.9)	1.9 (0.1, 5.8)
Pará	39300 (20900, 62900)	1270 (0, 8030)	40500 (22000, 66500)	4680 (695, 12700)	14.4 (7.7, 23.1)	0.5 (0, 3)	14.9 (8.1, 24.5)	1.7 (0.3, 4.7)
Pernambuco	36500 (18000, 62900)	999 (0, 6940)	37000 (18500, 63200)	6810 (1270, 16300)	14.1 (7, 24.4)	0.4 (0, 2.7)	14.3 (7.2, 24.5)	2.6 (0.5, 6.3)
Piauí	10000 (0, 23000)	313 (0, 2780)	9770 (0, 23300)	1890 (44, 6350)	11.2 (0, 25.6)	0.3 (0, 3.1)	10.9 (0, 26)	2.1 (0, 7.1)
Rio Grande do Norte	12000 (0, 24200)	375 (0, 2980)	12100 (0, 22900)	1700 (42, 5210)	13.3 (0, 26.8)	0.4 (0, 3.3)	13.4 (0, 25.4)	1.9 (0, 5.8)
Rio Grande do Sul	25000 (8310, 51900)	834 (0, 7980)	25400 (8980, 52100)	3580 (0, 16500)	9.9 (3.3, 20.6)	0.3 (0, 3.2)	10.1 (3.6, 20.7)	1.4 (0, 6.5)
Rio de Janeiro	59500 (28200, 105000)	1640 (0, 9670)	59400 (27900, 105000)	9200 (485, 30100)	15.1 (7.2, 26.7)	0.4 (0, 2.5)	15 (7.1, 26.6)	2.3 (0.1, 7.6)
Rondônia	5570 (0, 11800)	142 (0, 1250)	5430 (0, 11700)	1510 (0, 6320)	11.1 (0, 23.5)	0.3 (0, 2.5)	10.9 (0, 23.5)	3 (0, 12.6)
Roraima	2690 (753, 5350)	65 (0, 723)	2810 (945, 5420)	244 (0, 1050)	13.5 (3.8, 26.7)	0.3 (0, 3.6)	14 (4.7, 27.1)	1.2 (0, 5.3)
Santa Catarina	14000 (4300, 30200)	300 (0, 3170)	13700 (3970, 27800)	2280 (0, 9670)	8.4 (2.6, 18)	0.2 (0, 1.9)	8.2 (2.4, 16.6)	1.4 (0, 5.8)
Sergipe	6850 (1560, 13200)	126 (0, 1030)	6780 (0, 13100)	998 (0, 3530)	10.9 (2.5, 20.9)	0.2 (0, 1.6)	10.8 (0, 20.7)	1.6 (0, 5.6)
São Paulo	106000 (50800, 183000)	2340 (0, 22000)	107000 (50100, 188000)	11800 (0, 50900)	9.8 (4.7, 16.8)	0.2 (0, 2)	9.9 (4.6, 17.2)	1.1 (0, 4.7)
Tocantins	3670 (0, 9810)	133 (0, 1170)	3580 (0, 9840)	885 (0, 2950)	7.9 (0, 21.1)	0.3 (0, 2.5)	7.7 (0, 21.2)	1.9 (0, 6.3)
Total	630000 (526000, 751000)	16200 (3350, 46600)	635000 (534000, 758000)	91800 (53700, 150000)	11.8 (9.8, 14)	0.3 (0.1, 0.9)	11.9 (10, 14.2)	1.7 (1, 2.8)

Supplementary Table 7: Estimates of the number of children that lost a co-residing grandparent or elderly kin due to COVID-19 associated causes. Single refers to the loss of a single co-residing grandparent or elderly kin, multiple refers to the loss of more than 1, any refers to the loss of one or multiple, and only refers to the loss of a co-residing grandparent or elderly kin where there were no adults aged 18-64 in the household.

Region	Single	Multiple	Any	Only	Single (per 1000)	Multiple (per 1000)	Any (per 1000)	Only (per 1000)
Acre	741 (0, 2810)	6 (0, 0)	720 (0, 2690)	155 (0, 953)	2.4 (0, 9)	0 (0, 0)	2.3 (0, 8.6)	0.5 (0, 3)
Alagoas	2810 (53, 10500)	6 (0, 0)	2860 (127, 10000)	632 (0, 4350)	2.9 (0.1, 10.8)	0 (0, 0)	2.9 (0.1, 10.3)	0.6 (0, 4.5)
Amapá	934 (0, 3610)	2 (0, 0)	1020 (0, 4190)	101 (0, 867)	3.2 (0, 12.4)	0 (0, 0)	3.5 (0, 14.4)	0.3 (0, 3)
Amazonas	8750 (2440, 19800)	90 (0, 925)	8620 (2770, 18300)	1010 (0, 4470)	6.1 (1.7, 13.8)	0.1 (0, 0.6)	6 (1.9, 12.7)	0.7 (0, 3.1)
Bahia	6130 (0, 29700)	23 (0, 0)	6030 (0, 30200)	1280 (0, 12300)	1.6 (0, 7.6)	0 (0, 0)	1.6 (0, 7.8)	0.3 (0, 3.2)
Ceará	6410 (1070, 16700)	47 (0, 210)	6430 (895, 18700)	938 (0, 5810)	2.7 (0.4, 7)	0 (0, 0.1)	2.7 (0.4, 7.8)	0.4 (0, 2.4)
Distrito Federal	2320 (0, 8180)	12 (0, 0)	2230 (0, 7790)	53 (0, 652)	3.1 (0, 11)	0 (0, 0)	3 (0, 10.5)	0.1 (0, 0.9)
Espírito Santo	1970 (0, 7870)	2 (0, 0)	1950 (0, 6840)	205 (0, 1850)	1.9 (0, 7.7)	0 (0, 0)	1.9 (0, 6.7)	0.2 (0, 1.8)
Goiás	4610 (0, 16200)	32 (0, 0)	4670 (0, 18200)	1060 (0, 8010)	2.5 (0, 8.8)	0 (0, 0)	2.5 (0, 9.9)	0.6 (0, 4.4)
Maranhão	6800 (1340, 17100)	13 (0, 0)	6980 (1580, 17500)	1190 (0, 5190)	3 (0.6, 7.6)	0 (0, 0)	3.1 (0.7, 7.8)	0.5 (0, 2.3)
Mato Grosso	2990 (0, 10700)	12 (0, 0)	3060 (0, 12900)	410 (0, 3760)	3 (0, 10.9)	0 (0, 0)	3.1 (0, 13.2)	0.4 (0, 3.8)
Mato Grosso do Sul	2050 (0, 7490)	7 (0, 0)	2070 (0, 7930)	377 (0, 2450)	2.7 (0, 9.8)	0 (0, 0)	2.7 (0, 10.4)	0.5 (0, 3.2)
Minas Gerais	9040 (113, 30100)	56 (0, 0)	9510 (60, 36300)	1310 (0, 10000)	1.9 (0, 6.2)	0 (0, 0)	2 (0, 7.4)	0.3 (0, 2.1)
Paraná	6520 (245, 22300)	15 (0, 0)	6260 (165, 21500)	1010 (0, 6570)	2.3 (0.1, 8)	0 (0, 0)	2.3 (0.1, 7.7)	0.4 (0, 2.4)
Paraíba	2860 (27, 8710)	5 (0, 0)	2910 (78, 10400)	407 (0, 2750)	2.7 (0, 8.3)	0 (0, 0)	2.8 (0.1, 9.9)	0.4 (0, 2.6)
Pará	9030 (1800, 23600)	50 (0, 570)	9110 (1850, 23800)	1300 (0, 7350)	3.3 (0.7, 8.7)	0 (0, 0.2)	3.4 (0.7, 8.8)	0.5 (0, 2.7)
Pernambuco	6180 (410, 19600)	12 (0, 0)	6480 (503, 20100)	1260 (0, 7460)	2.4 (0.2, 7.6)	0 (0, 0)	2.5 (0.2, 7.8)	0.5 (0, 2.9)
Piauí	2550 (69, 9760)	2 (0, 0)	2730 (0, 10900)	408 (0, 3490)	2.8 (0.1, 10.9)	0 (0, 0)	3 (0, 12.2)	0.5 (0, 3.9)
Rio Grande do Norte	1910 (0, 7720)	3 (0, 0)	1910 (0, 7270)	295 (0, 2290)	2.1 (0, 8.6)	0 (0, 0)	2.1 (0, 8.1)	0.3 (0, 2.5)
Rio Grande do Sul	4570 (0, 18900)	10 (0, 0)	4570 (0, 18800)	712 (0, 6450)	1.8 (0, 7.5)	0 (0, 0)	1.8 (0, 7.5)	0.3 (0, 2.6)
Rio de Janeiro	11800 (1140, 33900)	14 (0, 0)	11800 (1030, 34700)	2340 (0, 14700)	3 (0.3, 8.6)	0 (0, 0)	3 (0.3, 8.8)	0.6 (0, 3.7)
Rondônia	2100 (180, 6790)	14 (0, 30)	2040 (201, 6700)	572 (0, 3670)	4.2 (0.4, 13.6)	0 (0, 0.1)	4.1 (0.4, 13.4)	1.1 (0, 7.3)
Roraima	679 (38, 2360)	2 (0, 0)	692 (55, 2230)	68 (0, 523)	3.4 (0.2, 11.8)	0 (0, 0)	3.5 (0.3, 11.1)	0.3 (0, 2.6)
Santa Catarina	3240 (0, 12800)	19 (0, 0)	3430 (0, 13300)	618 (0, 5060)	1.9 (0, 7.6)	0 (0, 0)	2.1 (0, 7.9)	0.4 (0, 3)
Sergipe	1180 (0, 4850)	4 (0, 0)	1200 (0, 5410)	167 (0, 1510)	1.9 (0, 7.7)	0 (0, 0)	1.9 (0, 8.6)	0.3 (0, 2.4)
São Paulo	24900 (2440, 76700)	78 (0, 0)	24700 (3180, 68000)	3140 (0, 27500)	2.3 (0.2, 7.1)	0 (0, 0)	2.3 (0.3, 6.3)	0.3 (0, 2.5)
Tocantins	1440 (0, 5760)	3 (0, 0)	1460 (0, 5940)	254 (0, 2000)	3.1 (0, 12.4)	0 (0, 0)	3.1 (0, 12.8)	0.5 (0, 4.3)
Total	134000 (88900, 201000)	539 (0, 4130)	135000 (85900, 199000)	21300 (5870, 62900)	2.5 (1.7, 3.8)	0 (0, 0.1)	2.5 (1.6, 3.7)	0.4 (0.1, 1.2)

References

[1] Jamie Ponmattam, Andrew Stokes, Lucas Carvalho, et al. "Covid-19 and Excess Mortality in Brazil: Subnational Estimates for 2020 and 2021". In: *In preparation* (2024).

[2] *Sistema de Informação Sobre Nascidos Vivos – Sinasc - 1996 a 2020 - OPENDATASUS.* (Visited on 04/25/2023).

[3] *IBGE — Biblioteca — Detalhes — Estudo Complementar à Aplicação Da Técnica de Captura-Recaptura : Estimativas Desagregadas Dos Totais de Nascidos Vivos e Óbitos : 2020 / IBGE, Coordenação de População e Indicadores Sociais.* https://biblioteca.ibge.gov.br/index.php/biblioteca-catalogo?view=detalhes&id=2101978. (Visited on 09/19/2023).

[4] IBGE. *Tábua Completa de Mortalidade Para o Brasil [: Análises e Tabelas].* 1999.

[5] Sheila Rizzato Stopa, Célia Landmann Szwarcwald, Max Moura de Oliveira, et al. "Pesquisa Nacional de Saúde 2019: histórico, métodos e perspectivas". In: *Epidemiologia e Serviços de Saúde* 29 (Oct. 2020), e2020315. ISSN: 1679-4974, 2237-9622. DOI: 10.1590/S1679-49742020000500004. (Visited on 09/14/2023).

[6] Gabriel Assuncao, Luna Hidalgo, and Douglas Braga. *PNSIBGE: Downloading, Reading and Analyzing PNS Microdata.* June 2023. (Visited on 09/19/2023).

[7] *Population Projection — IBGE.* https://www.ibge.gov.br/en/statistics/social/population/18176-population-projection.html?edicao=21933. (Visited on 09/14/2023).

[8] Seth Flaxman, Lackson Kasonka, Lucie Cluver, et al. "List Child Dependents on Death Certificates". In: *Science* 380.6644 (May 2023), pp. 467–467. DOI: 10.1126/science.adh8784. (Visited on 04/03/2024).

[9] Joseph L.F. De Kerf. "The Interpolation Method of Sprague-Karup". In: *Journal of Computational and Applied Mathematics* 1.2 (June 1975), pp. 101–110. ISSN: 03770427. DOI: 10.1016/0771-050X(75)90027-3. (Visited on 09/14/2023).

[10] Tim Riffe. *DemoTools.* Sept. 2023. (Visited on 09/14/2023).

[11] Thomas Lumley. *Survey: Analysis of Complex Survey Samples.* May 2023. (Visited on 09/06/2023).
