# Supplementary material for: Regional and national estimates of children affected by all-cause and COVID-19-associated orphanhood and caregiver death in Brazil, by age and family circumstance: a modeling study
Source: Lancet Reg Health Am. 2025 Sep 29;51:101252. doi: 10.1016/j.lana.2025.101252 (PMC12513060; doi:10.1016/j.lana.2025.101252)
Supplement: Translated abstract [file mmc2.docx]

**Editorial disclaimer**

The translation of the Summary was submitted by the authors, and we reproduce it as supplied. It has not been peer reviewed. Our editorial processes have only been applied to the original version in English, which should serve as a reference for this manuscript.

**Introdução** A orfandade e a morte de um cuidador podem ter consequências graves para as crianças. Dados oportunos e precisos podem orientar a política, principalmente durante emergências de saúde como a COVID-19. O objetivo do nosso estudo é apresentar análises nacionais e subnacionais da orfandade devido a todas as causas de morte de cuidadores e das mortes de cuidadores associadas à COVID-19 no Brasil e comparar os resultados com dados administrativos personalizados.

**Métodos** Usamos dados do Brasil disponíveis publicamente para estimar o número de crianças brasileiras que sofreram perda de pais e cuidadores devido à todas as causas de morte e à COVID-19 em 2020-2021.

**Resultados** Estima-se que 1.300.000 (intervalo de incerteza de 95%, 1.190.000, 1.430.000) crianças no Brasil sofreram a perda de um ou múltiplos pais e/ou cuidadores que co-residiam no domicílio. Estima-se que 673.000 (652.000, 690.000) perderam um ou ambos os pais, dos quais 149.000 (144.000, 154.000) morreram devido à COVID-19; estima-se que 635.000 (534.000, 758.000) crianças perderam um avô co-residente ou outro parente, dos quais 135.000 (85.900, 199.000) morreram devido à COVID-19.

A orfandade variou substancialmente entre os estados, com a taxa de orfandade parental por todas as causas mais alta em Roraima, 17,5 (15,6, 20,6) por 1.000 crianças, e a mais baixa em Santa Catarina, 9,5 (8,7, 10,4) por 1.000 crianças. A orfandade associada à COVID-19 também foi distribuída de forma desigual, com Mato Grosso experimentando a maior taxa, 4,4 (3,9, 5,3) por 1,000 crianças, enquanto o Pará experimentou a menor taxa, 1,4 (1,2, 1,8) por 1.000 crianças.

Comparações com dados limitados dos cartórios de registro civil do Brasil e das declarações de óbito revisadas manualmente na cidade de Campinas encontraram uma distribuição demográfica semelhante da orfandade. No entanto, nossas estimativas sugerem que as fontes administrativas subestimam a orfandade.

**Interpretação** Nossos resultados destacam a extensão da orfandade no Brasil e as grandes desigualdades entre os estados. Comparações com dados administrativos mostram padrões e proporções temporais semelhantes de orfandade materna e paterna, mas magnitudes diferentes. Isso sugere que o fortalecimento dos sistemas de registro vitais pode colocar as crianças no centro das respostas de saúde pública em todo o mundo.
